# ﻿Four new hypogean species of the genus *Triplophysa* (Osteichthyes, Cypriniformes, Nemacheilidae) from Guizhou Province, Southwest China, based on molecular and morphological data

**DOI:** 10.3897/zookeys.1185.105499

**Published:** 2023-11-28

**Authors:** Tao Luo, Ming-Le Mao, Chang-Ting Lan, Ling-Xing Song, Xin-Rui Zhao, Jing Yu, Xing-Liang Wang, Ning Xiao, Jia-Jun Zhou, Jiang Zhou

**Affiliations:** 1 School of Karst Science, Guizhou Normal University, Guiyang 550001, Guizhou, China; 2 School of Life Sciences, Guizhou Normal University, Guiyang 550025, Guizhou, China; 3 Guiyang Healthcare Vocational University, Guiyang 550081, Guizhou, China; 4 Zhejiang Forest Resource Monitoring Center, Hangzhou 310020, Zhejiang, China; 5 Zhejiang Forestry Survey Planning and Design Company Limited, Hangzhou 310020, Zhejiang, China

**Keywords:** Diversity, karst cave, morphology, new species, taxonomy, *Triplophysa*

## Abstract

Recently described cave species of the genus *Triplophysa* have been discovered in southwestern China, suggesting that the diversity of the genus is severely underestimated and that there may be many undescribed species. In this work, four new species of the genus *Triplophysa* are described from southwestern Guizhou Province, China, namely *Triplophysacehengensis* Luo, Mao, Zhao, Xiao & Zhou, **sp. nov.** and *Triplophysarongduensis* Mao, Zhao, Yu, Xiao & Zhou, **sp. nov.** from Rongdu Town, Ceheng County, Guizhou, *Triplophysapanzhouensis* Yu, Luo, Lan, Xiao & Zhou, **sp. nov.** from Hongguo Town, Panzhou City, Guizhou, and *Triplophysaanlongensis* Song, Luo, Lan, Zhao, Xiao & Zhou, **sp. nov.** from Xinglong Town, Anlong County, Guizhou. These four new species can be distinguished from all recognized congeners by a combination of morphological characteristics and significant genetic divergences. The discovery of these species increases the number of known cave species within the genus *Triplophysa* to 39, making the genus the second most diverse group of cave fishes in China after the golden-line fish genus *Sinocyclocheilus*. Based on the non-monophyletic relationships of the different watershed systems in the phylogenetic tree, this study also discusses the use of cave species of the genus *Triplophysa* to determine the possible historical connectivity of river systems.

## ﻿Introduction

The Southwest China Karst (SCK) topography is widely distributed and varied, with its extremely high level of biodiversity making the region a priority area for biological conservation in China (Ministry of Environmental Protection 2015). In the SCK, cave fishes, as representative organisms adapted to the dark environment of caves, are extremely diverse and show certain adaptations, including augmentation of non-visual senses (olfaction), more efficient metabolism (accumulation of fat), and structural or functional loss of specialized organs (loss or degradation of eyes, pigmentation, and circadian rhythmicity) (Borowsky 2018; Ma et al. 2019). Geology and paleoclimate play an important role in driving cave fishes biodiversity formation through geographic isolation (Yan 2017; Wen et al. 2022). As a result, a combination of biotic and abiotic factors may have led to an ancestral species showing a gradual increase in genetic divergence, resulting in the evolution of a species complex or of cryptic species that are morphologically similar but significantly genetically divergent (Xu and Che 2019). The discovery of new species in the SCK in the last decade also suggests the possibility of undescribed species within widely distributed species, especially in the genera *Triplophysa*, *Oreonectes*, *Troglonectes*, and *Yunnanilus* of the family Nemacheilidae (Wu et al. 2023).

The genus *Triplophysa* Rendahl, 1933 is a large group of smaller loaches distributed on the Qinghai-Tibetan Plateau and adjacent regions and currently containing over 180 recognized valid species (Zhang et al. 2020; Fricke et al. 2023). Morphological characteristics that distinguish this genus from other genera in the family Nemacheilidae include anterior and posterior nostrils being closely set; the posterior wall of the bony capsule of the swim bladder is present; there is a specific type of secondary sex characteristics in which males have tubercle-bearing, elevated skin on both sides of the head, and a thickened tuberculated pad or agglomerations on the dorsal surfaces of the broadened and widened pectoral-fin rays (Zhu 1989; Zheng et al. 2009; Prokofiev 2010; He et al. 2012; Ren et al. 2012; Yang et al. 2012; Wu et al. 2018a; Chen and Peng 2019; Deng et al. 2022). Within their known range, these species inhabit lakes, rivers, and streams, including the cave streams of the SCK (Zhu 1989; Lan et al. 2013). The wide distribution of species within the genus *Triplophysa*, the habitat diversity, and small body size may have combined to drive the evolution of diverse habits and life history traits, on the basis of which *Triplophysa* can be divided into two life groups: an epigean group and a hypogean or cave-dwelling group (Liu et al. 2022; Lu et al. 2022). Based on the level of adaptation to the cave environment, the hypogean group can be further subdivided into two morphological types, i.e., stygobionts and stygophiles (Zhao et al. 2011; Lu et al. 2022). In China, *Triplophysa* contains 102 species, of which 35 hypogean species are distributed in Chongqing, Guangxi, Guizhou, Yunnan, and Hunan provinces (Table 1) (Lan et al. 2013; Zhang et al. 2020; Chen et al. 2021; Liu et al. 2022; Lu et al. 2022), primarily in the Hongshui River (12 species), the Nanpanjiang River (nine species), the Wujiang River (six species), the Liujiang River (four species), the Beipanjiang River (one species), the Red River (one species), the Duliujiang River (one species), and the Yuanjiang River (one species) (see Table 1 and Suppl. material 2 for the detailed distribution).

**Table 1.** A list of 35 species of hypogean fishes of the genus *Triplophysa* distributed in the Southwest China Karst.

ID	Species	Province	Main drainage	Tributary	Reference
1	*T.aluensis* Li & Zhu, 2000	Yunnan	Pearl River	Nanpanjiang River	Li and Zhu 2000
2	*T.anshuiensis* Wu, Wei, Lan & Du, 2018	Guangxi	Pearl River	Hongshui River	Wu et al. 2018a
3	*T.baotianensis* Li, Liu & Li, 2018	Guizhou	Pearl River	Nanpanjiang River	Li et al. 2018
4	*T.erythraea* Liu & Huang, 2019	Hunan	Yangtze River	Wujiang River	Huang et al. 2019
5	*T.fengshanensis* Lan, 2013	Guangxi	Pearl River	Hongshui River	Lan et al. 2013
6	*T.flavicorpus* Yang, Chen & Lan, 2004	Guangxi	Pearl River	Hongshui River	Yang et al. 2004
7	*T.gejiuensis* (Chu & Chen, 1979)	Yunnan	Pearl River	Nanpanjiang River	Chu and Chen 1979
8	*T.guizhouensis* Wu, He & Yang, 2018	Guizhou	Pearl River	Hongshui River	Wu et al. 2018b
9	*T.huapingensis* Zheng, Yang & Che, 2012	Guangxi	Pearl River	Hongshui River	Zheng et al. 2012
10	*T.langpingensis* Yang, 2013	Guangxi	Pearl River	Hongshui River	Lan et al. 2013
11	*T.longipectoralis* Zheng, Du, Chen & Yang, 2009	Guangxi	Pearl River	Liujiang River	Zheng et al. 2009
12	*T.longliensis* Qiu, Yang & Chen, 2012	Guizhou	Pearl River	Hongshui River	Qiu et al. 2012
13	*T.luochengensis* Li, Lan, Chen & Du, 2017	Guangxi	Pearl River	Hongshui River	Li et al. 2017a
14	*T.macrocephala* Yang, Wu & Yang, 2012	Guangxi	Pearl River	Liujiang River	Yang et al. 2012
15	*T.nandanensis* Lan, Yang & Chen, 1995	Guangxi	Pearl River	Hongshui River	Lan et al. 1995
16	*T.nanpanjiangensis* Zhu & Cao, 1988	Yunnan	Pearl River	Nanpanjiang River	Zhu and Cao 1988
17	*T.nasobarbatula* Wang & Li, 2001	Guizhou	Pearl River	Liujiang River	Wang and Li 2001
18	*T.posterodorsalus* (Li, Ran & Chen, 2006)	Guangxi	Pearl River	Liujiang River	Ran et al. 2006
19	*T.qingzhenensis* Liu, Zen, & Gong, 2022	Guizhou	Yangtze River	Wujiang River	Liu et al. 2022
20	*T.qini* Deng, Wang & Zhang, 2022	Chongqing	Yangtze River	Wujiang River	Deng et al. 2022
21	*T.qiubeiensis* Li & Yang, 2008	Yunnan	Pearl River	Nanpanjiang River	Li et al. 2008
22	*T.rosa* Chen & Yang, 2005	Chongqing	Yangtze River	Wujiang River	Chen and Yang 2005
23	*T.sanduensis* Chen & Peng, 2019	Guizhou	Pearl River	Duliujiang River	Chen and Peng 2019
24	*T.shilinensis* Chen,Yang & Xu, 1992	Yunnan	Pearl River	Nanpangjiang River	Chen et al. 1992
25	*T.tianeensis* Chen, Cui & Yang, 2004	Guangxi	Pearl River	Hongshui River	Chen et al. 2004
26	*T.tianlinensis* Li, Li, Lan & Du, 2017	Yunnan	Pearl River	Hongshui River	Li et al. 2017b
27	*T.tianxingensis* Yang, Li & Chen, 2016	Yunnan	Pearl River	Nanpangjiang River	Yang et al. 2016
28	*T.wudangensis* Liu, Zen & Gong, 2022	Guizhou	Yangtze River	Wujiang River	Liu et al. 2022
29	*T.wulongensis* Chen, Sheraliev, Shu & Peng, 2021	Chongqing, Guizhou	Yangtze River	Wujiang River	Chen et al. 2021; this study
30	*T.xiangshuingensis* Li, 2004	Yunnan	Pearl River	Nanpanjiang River	Li 2004
31	*T.xiangxiensis* Yang, Yuan & Liao, 1986	Hunan	Yangtze River	Yuanjiang River	Yang et al. 1986
32	*T.xichouensis* Liu, Pan, Yang & Chen, 2017	Yunnan	Red River	Red River	Liu et al. 2017
33	*T.xuanweiensis* Lu, Li, Mao & Zhao, 2022	Yunnan	Pearl River	Hongshui River	Lu et al. 2022
34	*T.yunnanensis* Yang, 1990	Yunnan	Pearl River	Nanpanjiang River	Chu and Chen 1990
35	*T.zhenfengensis* Wang & Li, 2001	Guizhou	Pearl River	Beipanjiang River	Wang and Li 2001

**Figure 1.**
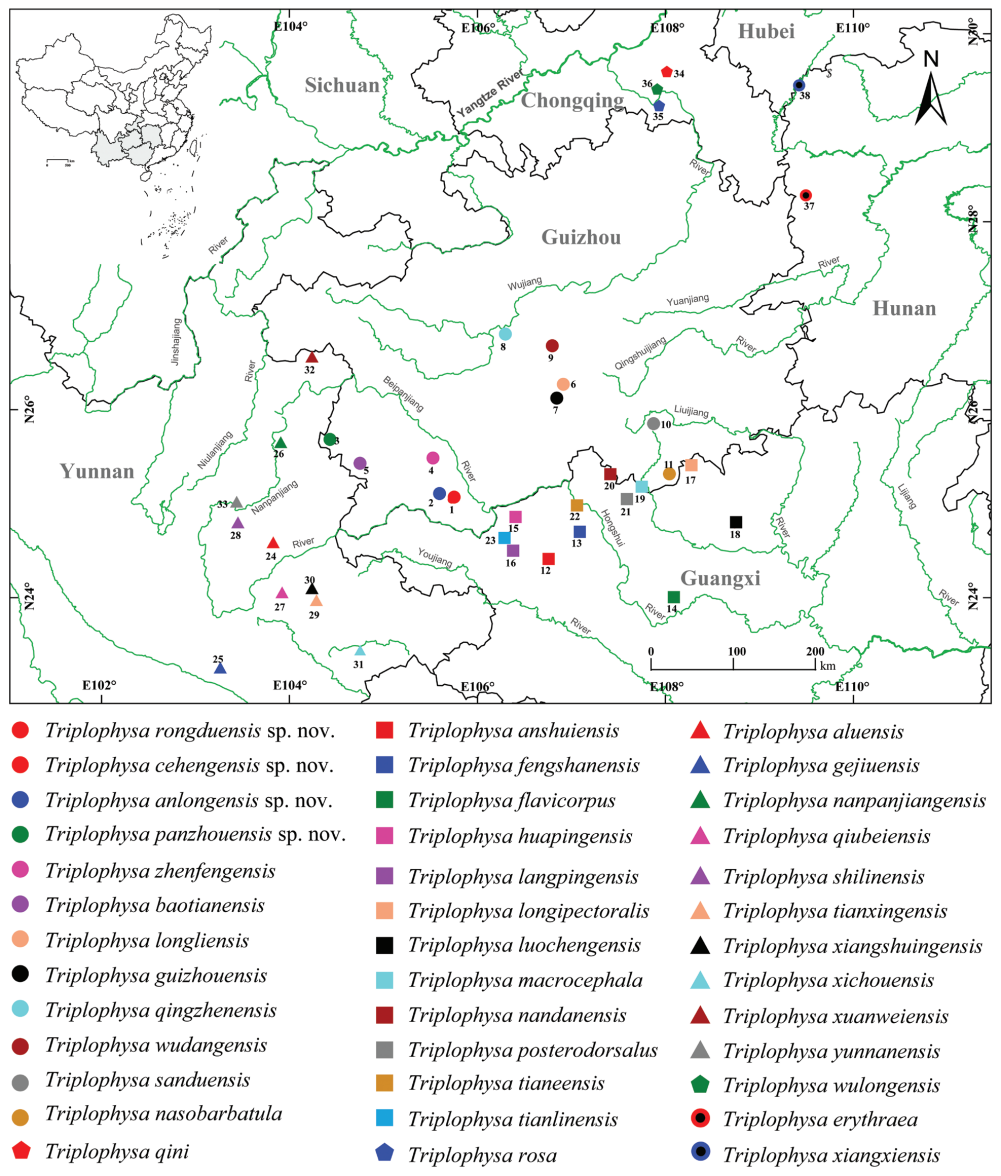
Sampling collection localities and distributions of the four new species and 35 hypogean species of the genus *Triplophysa* in southern China. For details concerning the ID numbers, please see Suppl. material 2. The base maps are from the Standard Map Service website (http://bzdt.ch.mnr.gov.cn/index.html).

Guizhou province is located in southwest China adjacent to Guangxi, Yunnan, Chongqing, and Sichuan provinces and is home to extensive karsts and well-developed underground rivers as well as being recognized as a cavefish biodiversity hotspot and a priority biodiversity reserve in China (Zhao and Zhang 2009; Ministry of Environmental Protection 2015). In the last decade, 16 cavefish species have been described from this region alone (Table 1). Currently, eight hypogean species of the genus *Triplophysa* have been recorded in Guizhou Province, i.e., *T.baotianensis* Li, Liu & Li, 2018 (Nanpanjiang River), *T.guizhouensis* Wu, He & Yang, 2018 (Hongshui River), *T.longliensis* Qiu, Yang & Chen, 2012 (Hongshui River), *T.nasobarbatula* Wang & Li, 2001 (Liujiang River), *T.qingzhenensis* Liu, Zen & Gong, 2022, *T.wudangensis* Liu, Zen & Gong, 2022 (Wujiang River), *T.sanduensis* Chen & Peng, 2019 (Duliujiang River), and *T.zhenfengensis* Wang & Li, 2001 (Beipanjiang River) (Wang and Li 2001; Qiu et al. 2012; Li et al. 2018; Wu et al. 2018a; Chen and Peng 2019; Liu et al. 2022). However, the current scattered distribution of these cave species in Guizhou indicates that the region has not been completely surveyed and that new species may still remain undiscovered (Fig. 1).

In January 2023, we collected several specimens of the genus *Triplophysa*, identified by the anterior and posterior nostrils being closely set, during a survey of cave fishes in southwestern Guizhou Province, China. Morphological examination and molecular phylogenetic analysis suggested that these specimens could be distinguished from the 35 hypogean species of the genus *Triplophysa*. Here, we formally describe the specimens as four new species, *Triplophysacehengensis* sp. nov., *Triplophysarongduensis* sp. nov., *Triplophysapanzhouensis* sp. nov., and *Triplophysaanlongensis* sp. nov.

## ﻿Materials and methods

### ﻿Sampling

In total, 73 samples from 20 species were collected for morphology comparison and genetic analysis (Fig. 1, Appendix 1). Among these were three specimens representing a new species, *Triplophysacehengensis* sp. nov., from Rongdu Town, Ceheng County, Guizhou; six specimens representing a new species, *Triplophysarongduensis* sp. nov., from Rongdu Town, Ceheng County, Guizhou; six specimens representing a new species, *Triplophysapanzhouensis* sp. nov., from Hongguo Town, Panzhou City, Guizhou, and six specimens representing a new species, *Triplophysaanlongensis* sp. nov., from Xinglong Town, Anlong County, Guizhou, China. Four specimens were identified as *T.baotianensis* from Baotian Town, Panzhou City, Guizhou; six specimens were *T.nasobarbatula* from Dongtang Township, Libo County, Guizhou; five specimens were *T.zhenfengensis* from Xinlongchang Town, Xingren County, Guizhou; six specimens were *T.guizhouensis* from Baijin Town, Huishui County, Guizhou; eight specimens were *T.macrocephala* Yang, Wu & Yang, 2012 from Lihu Town, Nandan County, Guangxi; two specimens were *T.langpingensis* Zheng, Yang & Che, 2012 from Langping Town, Tanlin County, Guangxi; seven specimens were *T.tianeensis* Chen, Cui & Yang, 2004 from Bala Township, Tian’e County, Guangxi; seven specimens were *T.huapingensis* Zheng, Yang & Che, 2012 from Huaping Township, Leye County, Guangxi; two specimens were *T.nandanensis* Lan, Yang & Chen, 1995 from Liuzhai Town, Nandan County, Guangxi; two specimens were *T.qiubeiensis* Li & Yang, 2008 from Nijiao Town, Qiubei County, Yunan; eight specimens were *T.qingzhenensis* from Qingzhen County, Guiyang City, Guizhou; nine specimens were *T.rosa* Chen & Yang, 2005 from Huolu Town, Wulong County, Chongqing; five specimens were *T.xiangxiensis* Yang, Yuan & Liao, 1986 from Feihu Cave, Hunan; five specimens were *T.qini* Deng, Wang & Zhang, 2022 from Dudu Village, Fengdu County, Chongqing; two specimens were *T.erythraea* Liu & Huang, 2019 from Dalong Cave, Huayuan County, Hunan, and one specimen was *T.wudangensis* from Wudang District, Guiyang City, Guizhou. All specimens were fixed in 10% buffered formalin and later transferred to 75% ethanol for preservation. Muscle samples used for molecular analysis were preserved in 95% alcohol and stored at −20 °C. All specimens were deposited at Guizhou Normal University (**GZNU**), Guiyang City, Guizhou Province, China.

### ﻿DNA extraction, PCR, and sequencing

Genomic DNA was extracted from muscle tissue using a DNA extraction kit from Tiangen Biotech (Beijing) Co. Ltd. In total, 12 tissue samples used for molecular analysis were amplified and sequenced for mitochondrial gene cytochrome *b* (Cyt *b*) using the primers L3975 (5’-CGCCTGTTTACCAAAAACAT-3’) and H4551 (5’-CCGGTCTGAACTCAGATCACGT-3’) following Xiao et al. (2001). PCR amplifications were performed in a 20 μl reaction volume with the following cycling conditions: an initial denaturing step at 95 °C for 4 min, 35 cycles of denaturing at 95 °C for 30 s, annealing at 52 °C for 1 min, and extension at 72 °C for 1 min followed by a final extension at 72 °C for 10 min. PCR products were puriﬁed with spin columns. The products were sequenced on an ABI Prism 3730 automated DNA sequencer at Chengdu TSING KE Biological Technology Co. Ltd. (Chengdu, China). All newly obtained sequences have been submitted to GenBank (Table 2).

**Table 2.** Localities, voucher information, and GenBank numbers for all samples used. Numbers in bold were generated in this study.

ID	Species	Localities (* type localities)	Voucher ID	Cyt *b*
1	*T.panzhouensis* sp. nov.	Hongguo Town, Panzhou City, Guizhou, China*	GZNU 20220513001	**OQ754119**
2	*T.panzhouensis* sp. nov.	Hongguo Town, Panzhou City, Guizhou, China*	GZNU 20220513002	**OQ754120**
3	*T.panzhouensis* sp. nov.	Hongguo Town, Panzhou City, Guizhou, China*	GZNU 20220513003	**OQ754121**
4	*T.cehengensis* sp. nov.	Rongdu Town, Ceheng County, Guizhou, China*	GZNU 20230109001	**OQ754132**
5	*T.cehengensis* sp. nov.	Rongdu Town, Ceheng County, Guizhou, China*	GZNU 20230109002	**OQ754133**
6	*T.cehengensis* sp. nov.	Rongdu Town, Ceheng County, Guizhou, China*	GZNU 20230109003	**OQ754134**
7	*T.rongduensis* sp. nov.	Rongdu Town, Ceheng County, Guizhou, China*	GZNU 20230110001	**OQ754135**
8	*T.rongduensis* sp. nov.	Rongdu Town, Ceheng County, Guizhou, China*	GZNU 20230110002	**OQ754136**
9	*T.rongduensis* sp. nov.	Rongdu Town, Ceheng County, Guizhou, China*	GZNU 20230110003	**OQ754137**
10	*T.anlongensis* sp. nov.	Xinglong Town, Anlong County, Guizhou, China*	GZNU 20230112001	**OQ754138**
11	*T.anlongensis* sp. nov.	Xinglong Town, Anlong County, Guizhou, China*	GZNU 20230112002	**OQ754139**
12	*T.anlongensis* sp. nov.	Xinglong Town, Anlong County, Guizhou, China*	GZNU 20230112003	**OQ754140**
13	*T.baotianensis*	Baotian Town, Panzhou City, Guizhou, China*	GZNU 20180421005	MT992550
14	*T.baotianensis*	Baotian Town, Panzhou City, Guizhou, China	GZNU 20180421006	**OQ241181**
15	*T.erythraea*	Dalong Cave, Huayuan County, Hunan, China*	/	MG967615
16	*T.huapingensis*	/	F3917	MG697589
17	*T.huapingensis*	Huaping Town, Leye County, Guangxi, China*	GZNU 20230404004	**OQ754125**
18	*T.langpingensis*	Longping Township, Tianlin County, Guangxi*	GZNU 20230404001	**OQ754122**
19	*T.longliensis*	/	SWU2016090300	MW582825
20	*T.macrocephala*	Lihu Town, Nandan County, Guangxi, China*	GZNU 20230404002	**OQ754123**
21	*T.nasobarbatula*	Dongtang Township, Libo County, Guizhou, China*	GZNU 20190114001	MH685911
22	*T.nasobarbatula*	Dongtang Township, Libo County, Guizhou, China*	GZNU 20220313010	**OQ241175**
23	*T.nasobarbatula*	Dongtang Township, Libo County, Guizhou, China*	GZNU 20220313011	**OQ241176**
24	*T.nandanensis*	Hechi City, Guangxi,China	SWU20151123046	MG697588
25	*T.nandanensis*	Liuzhai Town, Nandan County, Guangxi, China*	GZNU 20230404005	**OQ754126**
26	*T.nandanensis*	Liuzhai Town, Nandan County, Guangxi, China*	GZNU 20230404007	**OQ754128**
27	*T.qini*	Houping Village, Wulong County, Chongqing, China*		ON528184
28	*T.qiubeiensis*	NijiaoVillage, Qiubei County, Yunnan, China*	GZNU 20230404006	**OQ754127**
29	*T.qingzhenensis*	Qingzhen County, Guiyang City, Guizhou China*	IHB 201911150004	MT700458
30	*T.rosa*	Huolu Town, Wulong County, Chongqing, China	SWU10100503	JF268621
31	*T.rosa*	/	F3911	MG697587
32	*T.rosa*	Huolu Town, Wulong County, Chongqing City, China	GZNU 20230404009	**OQ754130**
33	*T.sanduensis*	Zhonghe Town, Sandu County, Guizhou, China*	SWU20170613001	MW582822
34	*T.tianeensis*	/	/	MW582826
35	*T.tianeensis*	Bala Township, Tian ‘e County, Guangxi, China*	GZNU 20230404003	**OQ754124**
36	*T.wudangensis*	Wudang District, Guiyang City, Guizhou Province, China*	IHB 201908090003	MT700460
37	*T.wudangensis*	Wudang District, Guiyang City, Guizhou Province, China*	GZNU 20230404010	**OQ754131**
38	*T.wulongensis*	Wulong County, Chongqing, China*	/	MW582823
39	*T.wulongensis*	Huolu Town, Wulong County, Chongqing City, China	GZNU 20230404008	**OQ754129**
40	*T.xiangxiensis*	Feihu Cave, Hunan, China*	/	JN696407
41	*T.xiangxiensis*	/	IHB 2015010002	KT751089
42	*T.xuanweiensis*	Tangtang Town, Xuanwei City, Yunnan, China*	ASIZB223818	OL675196
43	*T.xuanweiensis*	Tangtang Town, Xuanwei City, Yunnan, China*	ASIZB223819	OL675197
44	*T.xuanweiensis*	Tangtang Town, Xuanwei City, Yunnan, China*	ASIZB223820	OL675198
45	*T.zhenfengensis*	Xinlongchang Town, Xingren City, Guizhou, China*	GZNU 20220313007	**OQ241177**
46	*T.zhenfengensis*	Xinlongchang Town, Xingren City, Guizhou, China*	GZNU 20220313008	**OQ241178**
47	*T.zhenfengensis*	Xinlongchang Town, Xingren City, Guizhou, China*	GZNU 20220313009	**OQ241179**
48	*T.zhenfengensis*	Xinlongchang Town, Xingren City, Guizhou, China*	GZNU 20220313005	**OQ241180**
49	*T.nujiangensa*	Fugong County, Yunnan, China	IHB201315814	KT213598
50	*T.tibetana*	Mafamu lake, Xinjiang, China	NWIPB1106069	KT224364
51	*T.tenuis*	Niutou river, Qingshui County, Gansu, China	IHB0917490	KT224363
52	*T.wuweiensis*	Yongchang County, Gansu, China	IHB201307124	KT224365
53	*Barbatulabarbatula*	/	/	KP715096
54	*Barbatulalabiata*	Xinyuan County, Xinjiang, China	IHB201306569	KT192057
55	*Homatulaberezowskii*	Qujing City, Yunnan, China	FS-2014-Y03	NC_040302

### ﻿Phylogenetic analyses

In total, 55 mitochondrial Cyt *b* sequences were used for molecular analysis, including 22 that were newly sequenced and 33 that were downloaded from GenBank. We followed the phylogenetic study from Lu et al. (2022) and selected *Barbatulalabiata*, *B.barbatula*, and *Homatulaberezowskii* as outgroups.

All sequences were assembled and aligned using the MUSCLE (Edgar 2004) module in MEGA v. 7.0 (Kumar et al. 2016) with default settings. Alignment results were checked by eye. Phylogenetic trees were constructed via both maximum likelihood (ML) and Bayesian inference (BI) methods. The ML was conducted in IQ-TREE v. 2.0.4 (Nguyen et al. 2015) with 10000 ultrafast bootstrap (UBP) replicates (Hoang et al. 2018) and was performed until a correlation coefficient of at least 0.99 was reached. The BI was performed in MrBayes v. 3.2.1 (Ronquist et al. 2012), and the best-fit model was obtained based on the Bayesian information criterion computed with PartitionFinder v. 2.1.1 (Lanfear et al. 2017). In this analysis, the first, second and third codons of Cyt *b* were defined.

The analysis suggested the best partition scheme for each codon position of Cyt *b.* TRNEF+I+G, HKY+I, and TIM+I+G were selected for first, second, and third codons, respectively. Two independent runs were conducted in the BI analysis, each of which was performed for 2 × 10^7^ generations and sampled every 1000 generations. The first 25% of the samples were discarded as a burn-in, resulting in a potential scale reduction factor of < 0.01. Nodes in the trees were considered well supported when Bayesian posterior probabilities (BPP) were ≥ 0.95 and the ML ultrafast bootstrap value (UBP) was ≥ 95%. Uncorrected *p*-distances (1000 replicates) based on Cyt *b* were estimated using MEGA v. 7.0.

### ﻿Morphological comparisons

Morphometric data were collected from 36 well-preserved specimens of the genus *Triplophysa* (Suppl. material 3). A total of 37 measurements were recorded to the nearest 0.1 mm with digital calipers following the protocols of Tang et al. (2012) and Li et al. (2018). All measurements were taken on the left sides of the fish specimens. Measurements for the new species in this study are provided in Suppl. material 3.

Comparative data for the 35 hypogean species of the genus *Triplophysa* were obtained from the literature and specimen examination (Suppl. material 4). Specimens of 16 species from the type locality were collected and examined; these included *T.baotianensis*, *T.erythraea*, *T.guizhouensis*, *T.huapingensis*, *T.macrocephala*, *T.nandanensis*, *T.nasobarbatula*, *T.langpingensis*, *T.qingzhenensis*, *T.qini*, *T.qiubeiensis*, *T.rosa*, *T.tianeensis*, *T.wudangensis*, *T.xiangxiensis*, and *T.zhenfengensis* (see Appendix 1). Considering the morphological similarity, genetic differences, and geographical distances of the four new species to *T.huapingensis*, *T.baotianensis*, and *T.zhenfengensis*, the measurements were also included in the statistical analysis. Principal component analyses (PCAs) of size-corrected measurements and simple bivariate scatterplots were used to explore and characterize the morphometric differences between the new species and closely related species. Mann-Whitney *U* tests were used to determine the significance of differences in morphometric characteristics between the new species and similar species. All statistical analyses were performed using SPSS 21.0 (SPSS, Inc., Chicago, IL, USA), and differences were considered statistically significant at *P* < 0.05. PCAs of morphological data were performed after logarithmic transformation and under nonrotational conditions. In addition, as reported by other researchers (Parsons and Jones 2000; Polaszek et al. 2010), canonical discriminant analysis (CDA, George and Paul 2010) was used to classify individuals into different groups, where a priori membership was determined based on specimens belonging to different species. All pre-processing of morphological data was performed in Microsoft Excel (Microsoft Corporation 2016). Vertebrae were counted from X-ray scanned images provided by the Key Laboratory of Vertebrate Evolution and Human Origins, Institute of Vertebrate Paleontology and Paleoanthropology, Chinese Academy of Sciences. Skeletal counts refer to the previous methods described by Kumar et al. (2016) and Min et al. (2022). The identification of secondary sex characteristics followed previous research (Zhu and Cao 1988).

## ﻿Results

### ﻿Phylogenetic analyses and genetic divergence

**Figure 2.**
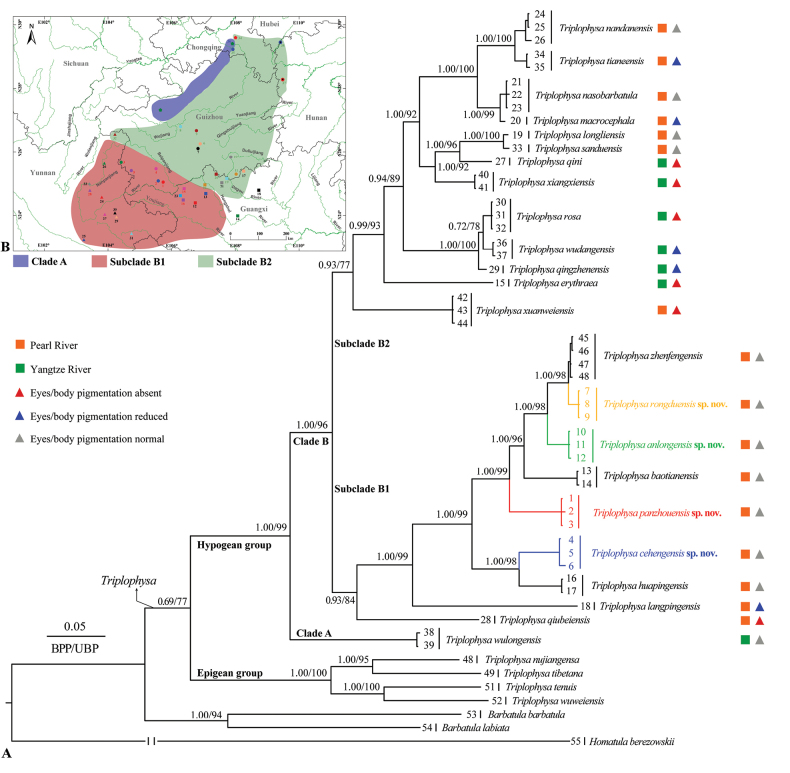
Phylogenetic tree based on **A** mitochondrial Cyt *b* (1140 bp) and **B** the corresponding ranges of the three clades. In this phylogenetic tree, Bayesian posterior probabilities (BPP) from BI analysis/ultrafast bootstrap supports (UBP) from ML analysis are noted beside nodes. Scale bars represent 0.05 nucleotide substitutions per site. The numbers at the tips of branches correspond to the ID numbers listed in Table 2.

Both ML and BI phylogenies were constructed based on mitochondrial Cyt *b* sequences, with the sequence length being 1140 base pairs. The BI and ML phylogenetic trees showed a highly consistent topology that strongly supported the monophyly of the genus *Triplophysa* and indicated that the genus could be divided into two major clades, the hypogean group and the epigean group (Fig. 2).

The hypogean group contained 19 known species from the karsts of southwestern China (Guizhou, Yunnan, Guangxi, Chongqing, and Hubei) and four other lineages from southwestern Guizhou that could be further divided into three clades (Fig. 2A): Clade A, only *T.wulongensis* Chen, Sheraliev, Shu & Peng, 2021, mainly in the Wujiang River basin (Fig. 2B); subclade B1, including *T.qiubeiensis*, *T.langpingensis*, *T.huapingensis*, *T.baotianensis*, *T.zhenfengensis*, and four other lineages from southwestern Guizhou, mainly in the Nampanjiang, Beipanjiang and Hongshui River basins (Fig. 2B); and subclade B2 including *T.xuanweiensis* Lu, Li, Mao & Zhao, 2022, *T.erythraea*, *T.qingzhenensis*, *T.wudangensis*, *T.rosa*, *T.xiangxiensis*, *T.qini*, *T.sanduensis*, *T.longliensis*, *T.macrocephala*, *T.nasobarbatula*, *T.tianeensis*, and *T.nandanensis*, mostly upstream of the Pearl and Yangtze rivers (Fig. 2B). The epigean group contained four known species from the Qinghai-Tibetan Plateau and adjacent regions, i.e., *T.wuweiensis* (Li & Chang, 1974), *T.tenuis* (Day, 1877), *T.tibetana* (Regan, 1905), and *T.nujiangensa* Chen, Cui & Yang, 2004 (Fig. 2).

Within subclade B1, all samples from Rongdu Town, Ceheng County, Guizhou (samples 4–6 in Table 2) clustered together in a sister clade to *T.huapingensis* with strong node support (BPP/UBP = 1.00/98). Also, this population could be distinguished from all known species and other undescribed lineages in this study by distinct morphological characteristics and molecular differences, with a lower *p*-distance of 6.1% (vs *T.huapingensis*) (Table 3). Thus, the population at this locality represents an independently evolved lineage and is described below as a new species, *Triplophysacehengensis* sp. nov.

**Table 3.** Uncorrected p-distance (%) between 23 species of the genus *Triplophysa* based on mitochondrial Cyt *b*.

ID	Species	1	2	3	4	5	6	7	8	9	10	11	12	13	14	15	16	17	18	19	20	21	22
1	*T.anlongensis* sp. nov.																						
2	*T.panzhouensis* sp. nov.	7.1																					
3	*T.cehengensis* sp. nov.	10.2	10.1																				
4	*T.rongduensis* sp. nov.	4.0	8.3	9.6																			
5	*T.baotianensis*	6.9	7.8	11.2	6.7																		
6	*T.erythraea*	14.7	13.7	15.4	14.7	14.2																	
7	*T.huapingensis*	10.1	10.1	6.1	9.3	10.6	15.4																
8	*T.longliensis*	14.8	13.2	14.1	14.3	14.6	11.0	15.0															
9	*T.langpingensis*	14.5	14.8	14.3	14.0	14.3	16.2	14.9	15.3														
10	*T.macrocephala*	15.5	15.2	15.5	15.4	15.9	12.0	15.1	9.5	15.8													
11	*T.nandanensis*	16.8	16.1	16.3	16.2	16.8	12.4	16.2	10.7	16.6	5.1												
12	*T.nasobarbatula*	15.6	15.3	15.4	15.2	15.8	12.1	15.0	9.5	15.2	0.7	5.3											
13	*T.qingzhenensis*	15.6	15.4	14.5	15.4	15.2	11.5	14.9	9.4	14.5	10.1	10.5	9.9										
14	*T.qiubeiensis*	14.6	14.4	14.6	14.0	14.3	13.8	14.7	13.6	14.6	14.5	14.9	14.1	13.9									
15	*T.qini*	14.8	14.4	14.1	14.6	15.5	10.8	15.3	5.4	15.8	9.3	10.7	9.5	9.1	13.5								
16	*T.rosa*	15.6	15.8	14.8	15.6	15.2	11.8	15.6	9.7	14.6	10.4	10.9	10.1	1.5	14.1	9.4							
17	*T.sanduensis*	15.0	13.6	14.1	14.5	14.8	11.2	15.0	0.5	15.3	9.4	10.8	9.4	9.4	13.8	5.5	9.7						
18	*T.tianeensis*	16.9	16.0	16.7	16.7	16.8	11.9	16.4	10.9	16.5	5.2	1.9	5.3	10.4	14.8	10.4	11.0	11.0					
19	*T.wudangensis*	15.4	15.6	14.7	15.4	14.9	11.5	15.0	9.6	14.5	10.5	10.8	10.2	1.6	14.3	9.6	1.5	9.7	10.8				
20	*T.wulongensis*	17.1	16.5	16.7	16.7	16.8	15.6	18.0	13.8	15.6	15.2	14.9	15.2	13.9	15.6	13.5	14.0	13.6	14.6	14.0			
21	*T.xiangxiensis*	14.5	14.3	14.6	14.3	15.1	11.7	15.6	7.7	14.9	8.5	10.0	8.4	9.0	14.1	6.0	9.0	8.0	9.3	9.2	14.7		
22	*T.xuanweiensis*	14.9	14.1	15.0	14.8	14.5	12.1	14.9	11.8	14.1	11.5	11.9	11.4	11.6	12.1	11.4	11.7	11.8	12.1	11.3	14.2	11.4	
23	*T.zhenfengensis*	3.5	8.0	9.4	0.8	6.9	14.8	9.3	14.7	13.9	15.7	16.6	15.5	15.7	14.1	14.9	15.9	14.9	17.0	15.8	16.6	14.6	14.6

Within subclade B1, all samples from Rongdu Town, Ceheng County, Guizhou (samples 7–9 in Table 2) clustered together in a sister clade to *T.zhenfengensis* with strong node support (BPP/UBP = 1.00/98). Moreover, this population could be distinguished from all known species and other undescribed lineages in this study by distinct morphological characteristics and molecular differences, with a lower *p*-distance of 0.8% (vs *T.zhenfengensis*). This is greater than the least genetic variation between recognized species, the 0.5% *p*-distance between *T.sanduensis* and *T.longliensis* (Table 3). Thus, the population at this locality represents an independently evolved lineage and is described below as a new species, *Triplophysarongduensis* sp. nov.

Within subclade B1, all samples from Xinglong Town, Anlong County, Guizhou (samples 10–12 in Table 2) clustered together in a sister clade to (*T.zhenfengensis* + *Triplophysarongduensis* sp. nov.) and with strong node support (BPP/UBP = 1.00/98). Also, this population could be distinguished from all known species and other undescribed lineages in this study by distinct morphological characteristics and molecular differences, with a lower *p*-distance of 3.5% (vs *T.zhenfengensis*) (Table 3). Thus, the population at this locality represents an independently evolved lineage and is described below as a new species, *Triplophysaanlongensis* sp. nov.

Within subclade B1, all samples from Hongguo Town, Panzhou City, Guizhou (samples 1–3 in Table 2) clustered together in a sister clade to (*T.baotianensis* + (*Triplophysaanlongensis* sp. nov. + (*T.zhenfengensis* + *Triplophysacehengensis* sp. nov.))) with strong node support (BPP/UBP = 1.00/99). Furthermore, this population could be distinguished from all known species and other undescribed lineages in this study by distinct morphological characteristics and molecular differences, with a lower *p*-distance of 7.8% (vs *T.baotianensis*) (Table 3). Thus, the population at this locality represents an independently evolved lineage and is described below as a new species, *Triplophysapanzhouensis* sp. nov.

### ﻿Morphological analyses

Mann-Whitney *U* tests and boxplots revealed differences in several morphological characteristics among the four new species (*T.cehengensis* sp. nov., *T.rongduensis* sp. nov., *T.panzhouensis* sp. nov., and *T.anlongensis* sp. nov.), and between the new species and the closely related species *T.baotianensis* and *T.zhenfengensis* (Suppl. materials 1, 5). In these pairwise morphometric comparisons, there were more significant differences between *T.cehengensis* sp. nov. and *T.panzhouensis* sp. nov. and between *T.cehengensis* sp. nov. and *T.zhenfengensis*, being 64.8% and 54.1%, respectively (Suppl. materials 1, 5). These significantly different measurements were concentrated on the head, eyes, fins, and tail. The differences were more pronounced in the new species with *T.cehengensis* sp. nov. and *T.panzhouensis* sp. nov., where 67.6% of the morphological characteristics were significantly different (*p*-values = 0.009−0.036) (Suppl. material 5).

**Figure 3.**
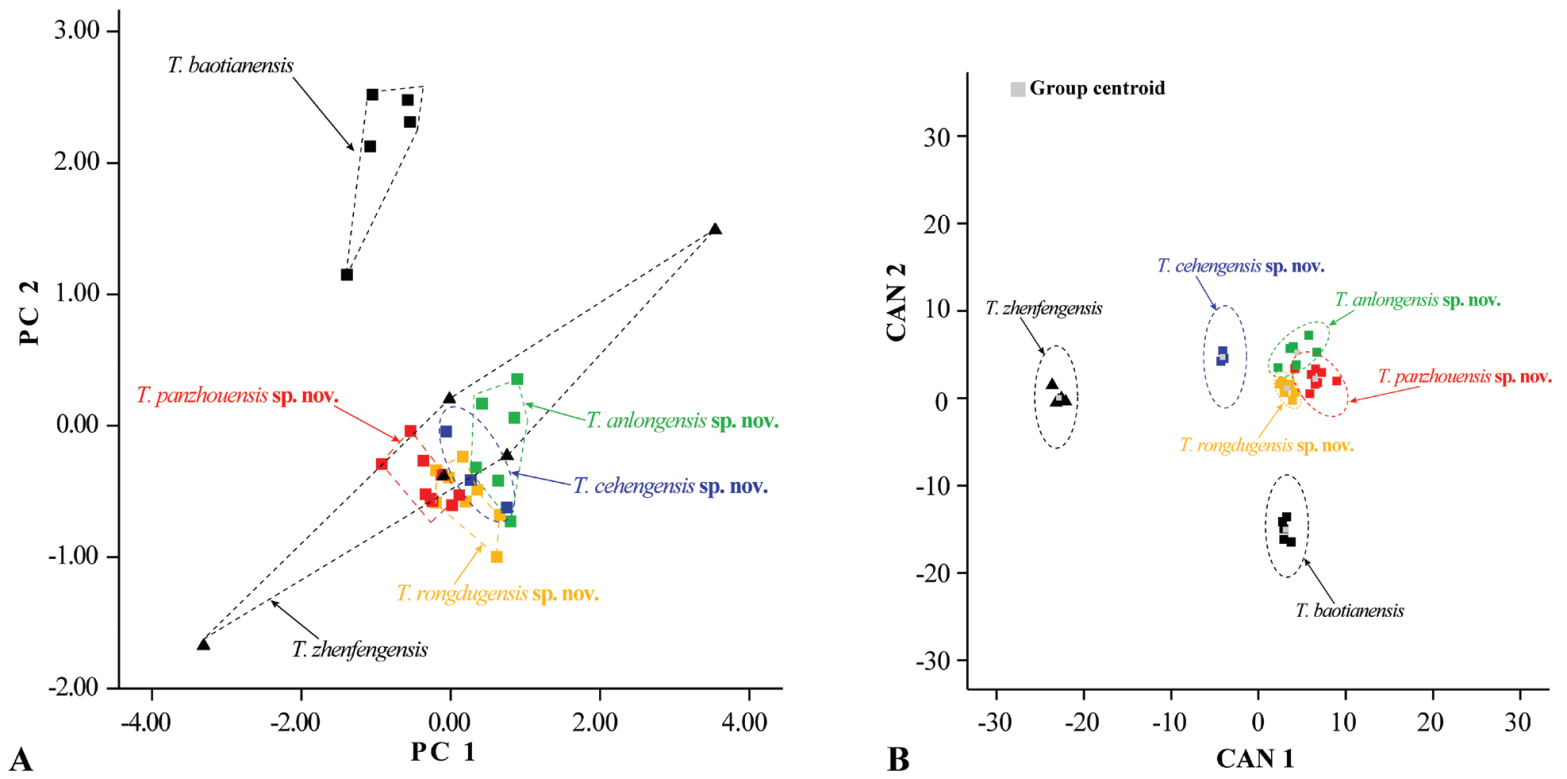
Plots of principal components (**A**) and canonical discriminant (**B**) analysis scores of *T.cehengensis* sp. nov., *T.rongduensis* sp. nov., *T.panzhouensis* sp. nov., *T.anlongensis* sp. nov., and closely related species based on morphometric data.

Seven principal component factors with eigenvalues greater than one were extracted based on PCA of the morphological data. These factors accounted for 84.05% of the total variation (Suppl. material 6). The first principal component (PC1) accounted for 35.41% of the variation and was positively correlated with all variables (eigenvalue = 18.86), which reflected the morphological differences between the four new species and similar species and corresponding to head length, head width, pre-anterior nostril length, distance between anterior nostrils, snout length, upper jaw length, pre-dorsal length, pectoral-fin base length, pre-pectoral length, and pre-pelvic length. The second principal component (PC2) accounted for 17.93% of the variation and was dominated by the distance between anterior and posterior nostrils, the distance between posterior nostrils, and mouth width (eigenvalue = 3.54) (Suppl. material 6). On the two-dimensional plots of PC1 and PC2, the four new species can be readily distinguished from *T.baotianensis* and *T.zhenfengensis*, but the four new species are closely clustered (Fig. 3A). CDA correctly classified 100% of the individuals in the initial grouping case for the six sample groups. Canonical axes (CAN) 1–2 explained 55.2% and 23.8% of the total variation (Fig. 3B, Suppl. material 6). On the two-dimensional plots of CAN1 and CAN2, the four new species are readily distinguishable from *T.baotianensis* and *T.zhenfengensis*, with *T.cehengensis* sp. nov. clearly separated from the remaining three species, while *T.rongduensis* sp. nov., *T.panzhouensis* sp. nov., and *T.anlongensis* sp. nov. are somewhat clustered, although they can be separated. Based on statistical analysis of the measurements and the PCA and CDA results, four new species are clearly distinguished in morphological characteristics from the geographical and morphological relatives *T.baotianensis* and *T.zhenfengensis*.

The hypogean group of the genus *Triplophysa* in this study can be phylogenetically divided into two clades A and B, where Clade B can be further subdivided into subclades B1 and B2. These clades can be defined by the following morphology: (1) for Clade A, the dorsal-fin origin posterior to the pelvic-fin origin; (2) for Clade B, the dorsal-fin origin anterior/opposite to the pelvic-fin origin and subclades B1 and B2 can be defined by the number of unbranched pelvic fin rays (two vs one). The four new species are based on the following characteristics: the dorsal fin origin anterior to the pelvic fin origin and the two unbranched pelvic-fin rays and can be easily placed into subclade B1. Thus, the new species can be compared to the five species within the remaining subclade B1 and the 15 species in the undefined clade.

### ﻿Taxonomic account

#### ﻿ Triplophysa cehengensis

Taxon classificationAnimaliaCypriniformesNemacheilidae

Luo, Mao, Zhao, Xiao & Zhou sp. nov.

E0D6C79A-3422-591A-B2F6-96FE7A2F3159

https://zoobank.org/21AF55DC-3FCA-439B-A15B-87296BEA10C8

**Figure 4.**
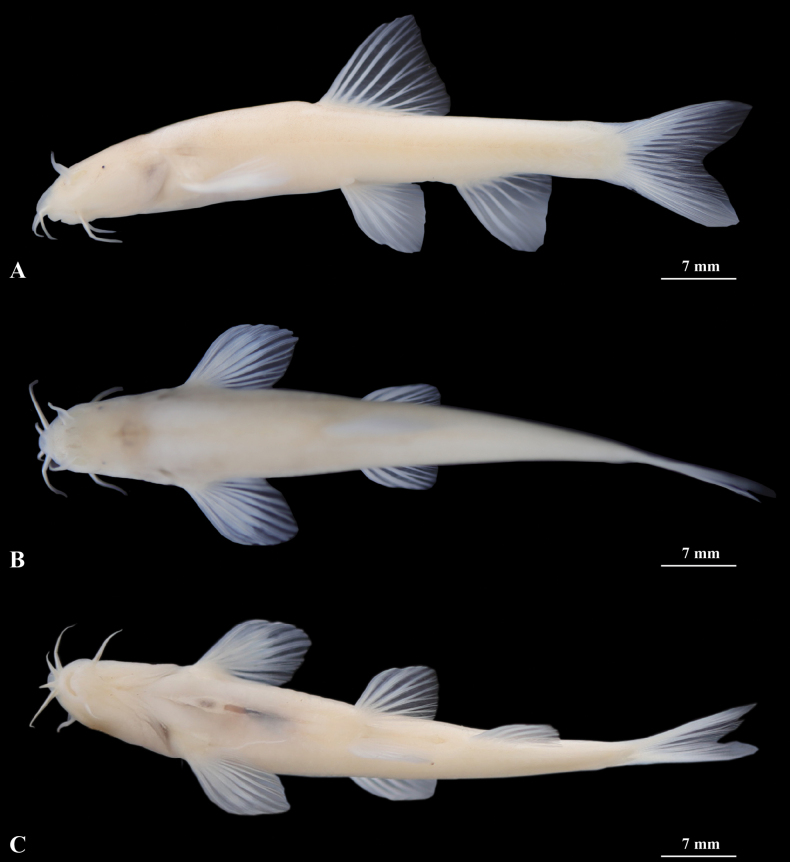
Morphological characteristics of holotype GZNU20230214010 of *Triplophysacehengensis* sp. nov. in preservative (10% formalin) **A** lateral view **B** dorsal view **C** ventral view. Photographs by Tao Luo.

**Figure 5.**
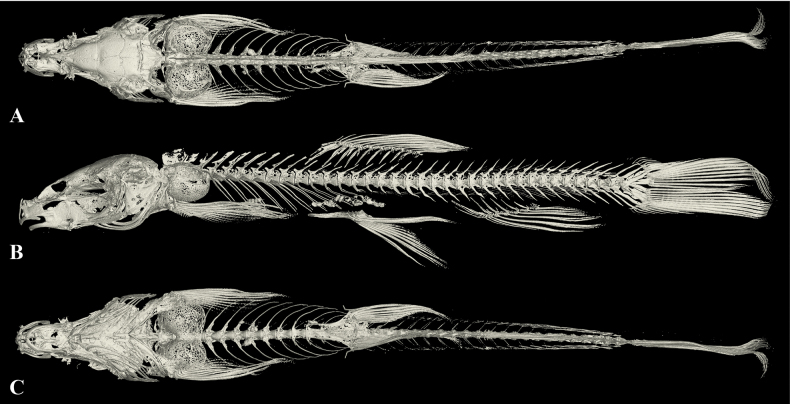
The three-dimensional reconstructed model of the skeleton of *Triplophysacehengensis* sp. nov. (paratype GZNU20230214011, standard length 38.6 mm) **A** dorsal view **B** lateral view and **C** ventral view.

**Figure 6.**
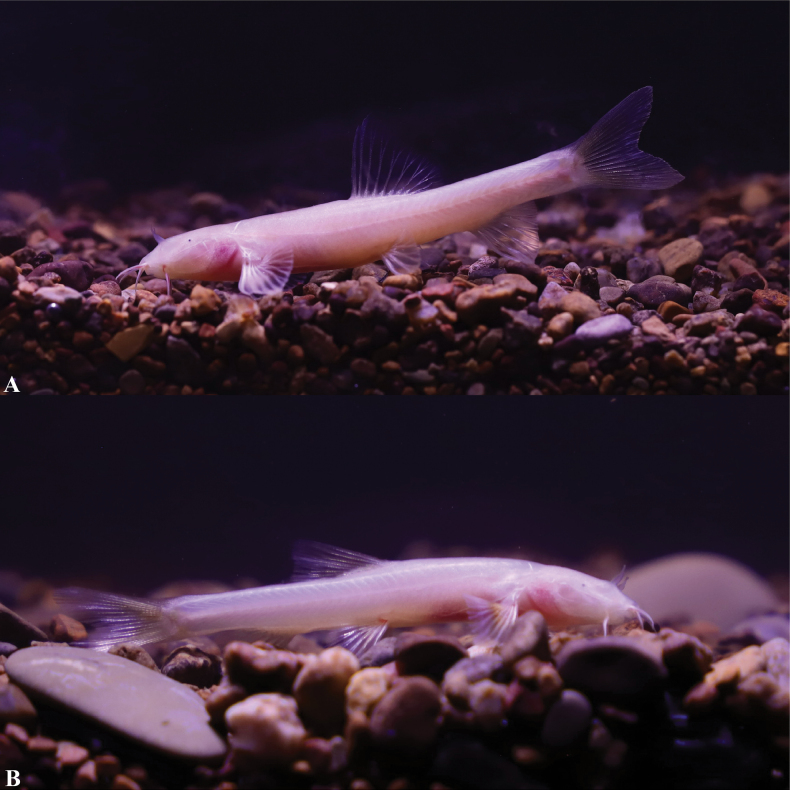
*Triplophysacehengensis* sp. nov. in life **A** holotype GZNU20230214010 **B** paratype GZNU20230214011.

Figs 4 , 5 , 6 , Suppl. materials 3 , 5

##### Type material.

***Holotype*.** GZNU20230214010 (Fig. 4), 63.76 mm total length (TL), 51.17 mm standard length (SL), collected by Tao Luo on January 7, 2023, at Longjing Village, Rongdu Town, Ceheng County, Guizhou Province, China (25.0410879°N, 105.72512627°E; ca. 1228 m. a.s.l.; Fig. 1).

***Paratypes*.** Two specimens from the same locality as the holotype: GZNU202302140011 and GZNU20230214012 collected by Tao Luo, Wei-Feng Wang, Xin-Rui Zhao, Jing Yu, and Chang-Ting Lan on January 7, 2023.

##### Diagnosis.

*Triplophysacehengensis* sp. nov. can be distinguished from all other congeners by the following combination of characteristics: (1) body naked, scaleless, without skin pigmentation; (2) eyes reduced, diameter 2% of head length (HL), interorbital width 27–37% of HL; (3) dorsal fin distal margin emarginated; (4) pelvic-fin tip reaching to anus; (5) tip of pectoral fin not reaching to pelvic fin origin; (6) anterior and posterior nostrils closely set, with the anterior nostril elongated to a barbel-like tip; (7) tip of outrostral barbel extending backward, not reaching to anterior margin of the eye; (8) lateral line complete; (9) dorsal-fin rays iv-9, pectoral-fin rays i-10, pelvic-fin rays ii-8, anal-fin rays iii-5, 16 branched caudal-fin rays; and (10) total vertebrae 39.

##### Description.

Morphological data of the specimens of the *Triplophysacehengensis* sp. nov. are provided in Suppl. materials 3, 5. Body elongated and cylindrical, posterior portion gradually compressed from dorsal fin to caudal-fin base, with deepest body depth anterior to dorsal-fin origin, deepest body depth 14–15% of standard length (SL). Dorsal profile slightly convex from snout to dorsal-fin insertion, then straight from posterior portion of dorsal-fin origin to caudal-fin base. Ventral profile flat. Head short, length 25–27% of SL, slightly depressed and flattened, width slightly greater than depth (head width (HW)/head depth (HD) = 1.13). Snout short, lightly blunt, length 47–54% of HL. Mouth inferior and curved, mouth corner situated below anterior nostril, upper and lower lips smooth, lips thick with shallow furrows, lower lip with a V-shaped median notch. Upper and lower jaw arched. Three pairs of barbels are present: inrostral barbel short, length 13–25% of HL, backward extending to corner of the mouth; outrostral barbel long, length 27–38% of HL, backward extending and not reaching to anterior margin of the eye. Maxillary barbel developed, length 19–36% of HL, tip of maxillary barbel reaching to anterior margin of operculum. Anterior and posterior nostrils closely set, length 0.2–0.5 mm, 3–4% of HL. Anterior nostril tube long, with an elongated short barbel-like tip, tip of posterior nostril extending backwards not reaching to anterior margin of the eye. Eyes reduced, with diameter ~ 2% of HL. Gill opening small, gill rakers not developed, nine inner gill rakers on first gill arch (*n* = 1).

Dorsal-fin rays iv-9, pectoral-fin rays i-10, pelvic-fin rays ii-8, anal-fin rays iii-5, 16 branched caudal-fin rays. Dorsal fin short, length 22–25% of SL, distal margin emarginated, origin anterior to pelvic-fin insertion and situated slightly posterior to the midpoint between snout tip and caudal-fin base, first branched ray longest, shorter than head length, tip of dorsal fin vertical to the anus. Pectoral fin moderately developed, length 21–23% of SL, tip of pectoral fin extending backward almost to the midpoint between origin of pectoral and pelvic fin origins, not reaching to pelvic fin origin. Pelvic fin length 17–18% of SL, vertically aligned with third branched ray of dorsal fin, tips of pelvic fin reaching to anus. Anal fin long, length 19–21% of SL, distal margin truncated, origin close to anus, tips of anal fin not reaching caudal-fin base, distance between tips of anal fin and anus 8.5× the eye diameter. Caudal fin forked, upper lobe is equal in length to lower lobe, tips pointed, caudal peduncle length 6.9 mm, caudal peduncle depth 3.5 mm, with weak adipose crests along both dorsal and ventral sides. Total vertebrae: 39 (*n* = 1).

Cephalic lateral line system developed. Lateral line complete, exceeding tip of pectoral fin and reaching base of caudal fin. Two chambers of air bladder, anterior chamber dumbbell-shaped and membranous, open on both sides, slightly closed posteriorly (Fig. 5); posterior chamber developed, slightly filling the body cavity, connected with anterior chamber by a long, slender tube.

##### Coloration.

In cave water bodies when alive, body semi-translucent and pale pink, without skin pigment, and all fins hyaline (Fig. 6). After fixation in 10% formalin solution, the body color was yellowish white, all fins transparent (Fig. 4).

##### Secondary sex characteristics.

No secondary sex characteristics was observed based on the present specimens of *Triplophysacehengensis* sp. nov.

##### Comparisons.

Comparative data of *Triplophysacehengensis* sp. nov. with the 34 recognized hypogean species within genus *Triplophysa* are given in Suppl. material 4. Based on the dorsal-fin origin anterior to the pelvic-fin origin and the unbranched pelvic-fin rays 2, new species can be placed within defined subclade B1. Thus, new species and morphological differences with subclade B1 and 15 undefined clade species were compared in detail.

*Triplophysacehengensis* sp. nov. can be distinguished from *T.baotianensis*, *T.zhenfengensis*, *T.huapingensis*, *T.flavicorpus*, *T.guizhouensis*, *T.longipectoralis*, *T.luochengensis*, *T.nanpanjiangensis*, *T.tianxingensis*, *T.xiangshuingensis*, *T.xichouensis*, and *T.yunnanensis* by body pigmentation absence (vs body pigmentation presence). *Triplophysacehengensis* sp. nov. can be distinguished from *T.qiubeiensis*, *T.anshuiensis*, *T.fengshanensis*, *T.gejiuensis*, *T.posterodorsalus*, and *T.shilinensis* by eyes reduced, diameter 2% of HL (vs eyes absent).

For the remaining two species, the new species share the following morphological characteristics: body pigmentation absent, eyes reduced, and body scaleless. However, *Triplophysacehengensis* sp. nov. differs from *T.aluensis and T.langpingensis* by the dorsal fin distal margin being emarginated (vs truncated); from *T.aluensis* by dorsal-fin rays (iv, 9 vs iii, 7), pelvic-fin rays (ii, 8 vs i, 6), 16 branched caudal-fin rays (vs 13), tip of pelvic fin reaching to anus (vs not reaching to anus); and from *T.langpingensis* by dorsal-fin rays (iv, 9 vs iii, 7–8), pelvic fin rays (ii, 8 vs i, 6), 16 branched caudal-fin rays (vs 14), lateral line complete (vs incomplete), and 16 branched caudal-fin rays (vs 14).

##### Ecology and distribution.

Currently, this new species *Triplophysacehengensis* sp. nov. has only been found in a cave in Longjing Village, Rongdu Town, Ceheng County, Guizhou Province, China, at an elevation of 1228 m. The pool where the new species was found to live is more than 30 cm long, 25 cm wide, and 6 cm deep, with a slow flow of water, and is located ~ 30 m vertically down from the cave entrance. Inside the cave, bats (*Iaio*, six individuals) and crabs (*Diyutamoncereum*, ten individuals) were found. Outside the cave, maize and rice are being grown. The population of the new species is very small. From a total of eight sample collections, only specimen number three was obtained.

##### Etymology.

The specific epithet *cehengensis* is in reference to the type locality of the new species: Longjing Village, Rongdu Town, Ceheng County. We propose the common English name “Ceheng high-plateau loach” and the Chinese name “Cè Hēng Gāo Yuán Qīu (册亨高原鳅)”.

#### ﻿ Triplophysa rongduensis

Taxon classificationAnimaliaCypriniformesNemacheilidae

Mao, Zhao, Yu, Xiao & Zhou sp. nov.

AF7553E4-0BA5-5714-BB38-DD6973FB646B

https://zoobank.org/D560BA11-E6EF-4BDE-AB94-985BA636AF35

**Figure 7.**
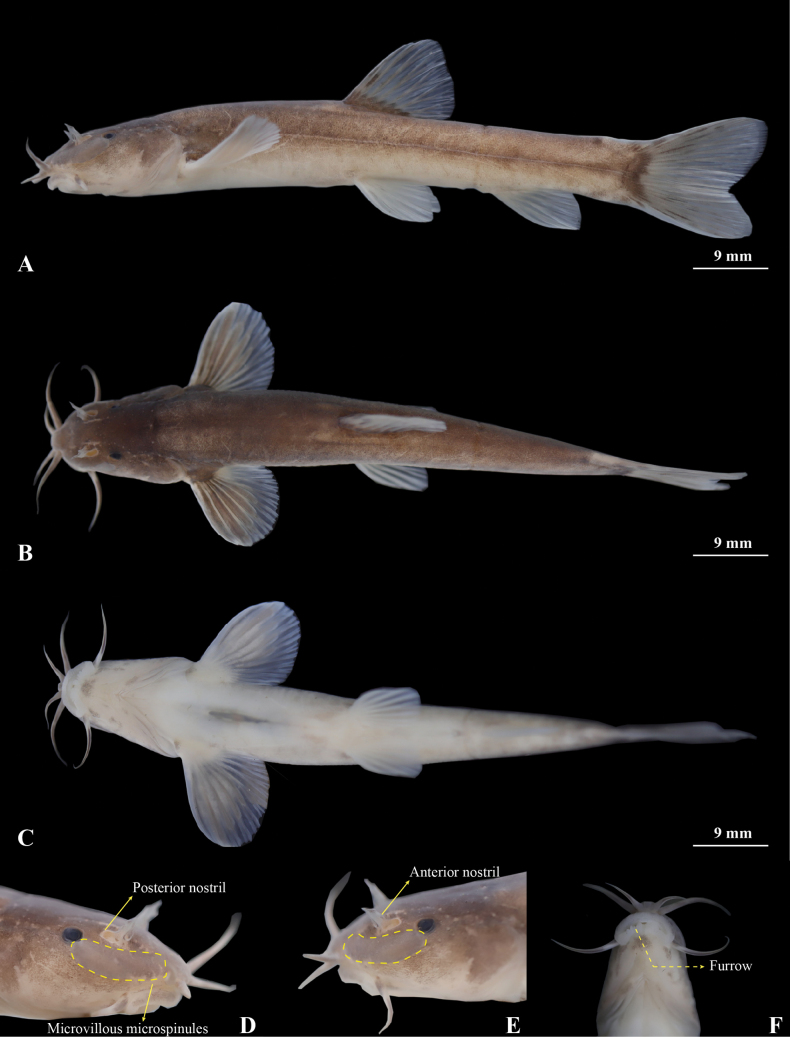
Morphological characteristics of holotype GZNU20230106001 of *Triplophysarongduensis* sp. nov. in preservative (10% formalin) **A** lateral view **B** dorsal view **C** ventral view **D** right side view of head **E** left side view of head, and **F** ventral view of head. Photographs by Tao Luo.

**Figure 8.**
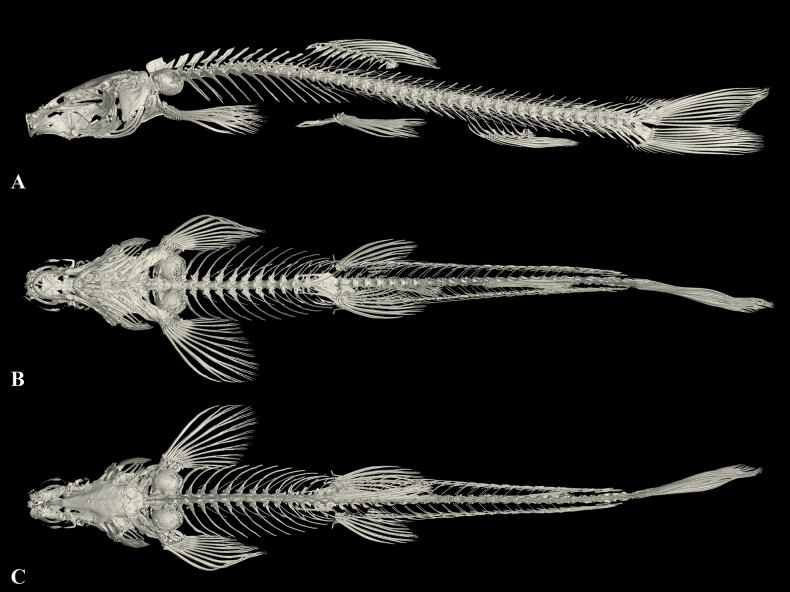
The three-dimensional reconstructed model of the skeleton of *Triplophysarongduensis* sp. nov (paratype GZNU20230223002, standard length 43.6 mm) **A** lateral view **B** ventral view, and **C** dorsal view.

**Figure 9.**
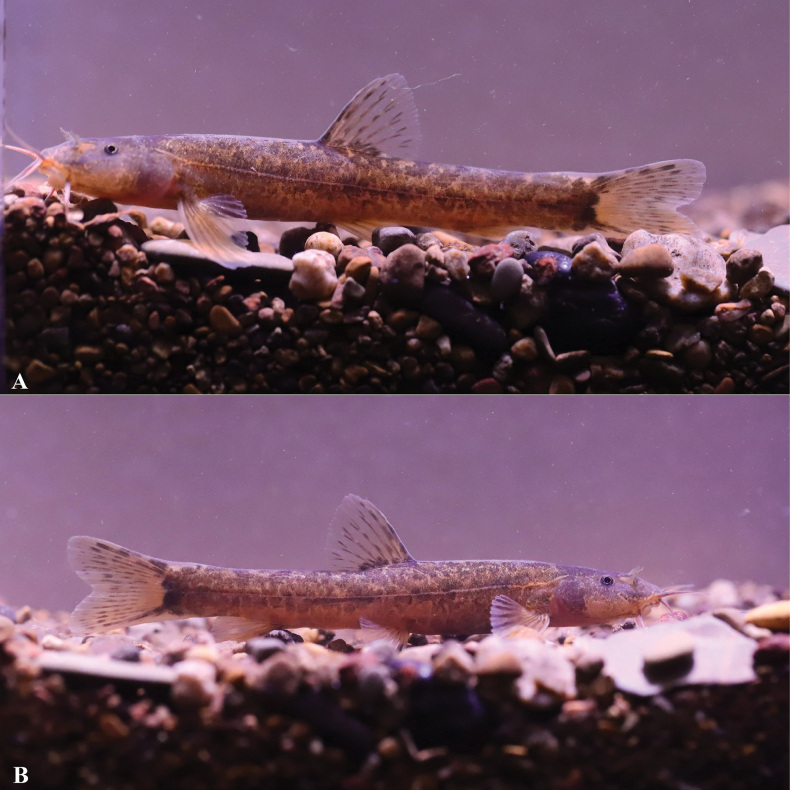
*Triplophysarongduensis* sp. nov. in life, paratype GZNU20230106001 **A** left side view **B** right side view.

Figs 7 , 8 , 9 , Suppl. materials 3 , 5

##### Type material.

***Holotype*.** GZNU20230106001 (Fig. 7), 84.7 mm total length (TL), 68.8 mm standard length (SL), collected by Tao Luo on January 6, 2023, in Rongbei Village, Rongdu Town, Ceheng County, Guizhou Province, China (25.04552008°N, 105.68482876°E; ca. 1173.2 m. a.s.l.; Fig. 1).

***Paratypes*.** Five specimens from the same locality as the holotype: GZNU20230214001–214005 collected by Tao Luo, Chang-Ting Lan, Jing Yu, Xin-Rui Zhao, and Wei-Feng Wang on January 6, 2023.

##### Diagnosis.

*Triplophysarongduensis* sp. nov. can be distinguished from species of the hypogean group of its congeners by the following combination of characteristics: (1) body naked, scaleless, with skin pigmentation; (2) eyes normal, diameter 7–15% of HL, interorbital width 24–32% of HL; (3) dorsal fin distal margin truncated; (4) pelvic-fin tip not reaching to anus; (5) tip of pectoral fin not reaching to pelvic fin origin; (6) anterior and posterior nostrils closely set, anterior nostril elongated to a barbel-like tip; (7) tip of outrostral barbel extending backward to posterior margin of the eye; (8) lateral line complete; (9) dorsal-fin rays iv-9, pectoral-fin rays i-10, pelvic-fin rays ii-7, anal-fin rays iii-5, 16 branched caudal-fin rays; and (10) total vertebrae 43.

##### Description.

Morphological data of the specimens of the *Triplophysarongduensis* sp. nov. are provided in Suppl. materials 3, 5. Body elongated and cylindrical, posterior portion gradually compressed from dorsal fin to caudal-fin base, with deepest body depth anterior to dorsal-fin origin, deepest body depth 13–16% of snout length. Dorsal profile slightly convex from snout to dorsal-fin insertion, then straight from posterior portion of dorsal-fin origin to caudal-fin base. Ventral profile flat. Head slightly long, length 24–26% of SL, head slightly depressed and flattened, width slightly greater than depth (HW/HD = 1.2). Snout short, slightly blunt, length 36–52% of HL. Mouth inferior and curved, mouth corner situated below anterior nostril, upper and lower lips smooth, lips thick with shallow furrows, lower lip with a V-shaped median notch. Processus dentiformis absent. Three pairs of barbels are present: inrostral barbel short, length 16–28% of HL, backward extending reaching to corner of mouth; outrostral barbel long, 40–54% of HL, backward extending to posterior margin of the eye. Maxillary barbel developed, length 30–43% of HL, tip of maxillary barbel reaching to anterior margin of operculum. Anterior and posterior nostrils closely set, length 0.4 mm. Anterior nostril tube long, with an elongated short barbel-like tip, elongated barbel-like tip of posterior nostril extending backwards not reaching the anterior margin of the eyes. Eyes present, normal, 7–15% of HL. Gill opening small, gill rakers not developed, eight inner gill rakers on the first gill arch (*n* = 1).

Dorsal-fin rays iv-9, pectoral-fin rays i-10, pelvic-fin rays ii-7, anal-fin rays iii-5, 16 branched caudal fin rays. Dorsal fin long, 19–25% of SL, distal margin emarginated, origin slightly anterior to pelvic-fin insertion, situated slightly posterior to the midpoint between snout tip and caudal-fin base; first branched ray longest, shorter than head length, tip of dorsal fin not extending to vertical of anus. Pectoral fin more developed, 18–20% of SL, tip of pectoral fin extends backwards not to the midpoint between origin of pectoral and pelvic fin origins, not reaching to pelvic fin origin. Pelvic fin length 7–17% of SL, vertically aligned with third branched ray of dorsal fin, tips of pelvic fin not reaching to anus, distance between tips of pelvic fin and anus 0.9× the eye diameter. Anal fin long, length 16–17% of SL, distal margin truncated, origin close to anus, spacing 2.1 mm, tips of anal fin not reaching caudal-fin base, distance between tip of anal fin and anus 1.5× the eye diameter. Caudal fin forked, upper lobe equal in length to lower lobe, tips pointed, caudal peduncle length 9.7 mm, caudal peduncle depth 6.0 mm, without adipose crests along both dorsal and ventral sides. Total vertebrae: 43 (*n* = 1).

Cephalic lateral line canals unclear. Lateral line complete and straight, exceeding tip of pectoral fin and reaching base of caudal fin. Two chambers of air bladder, anterior chamber dumbbell-shaped and membranous, open on both sides, slightly closed posteriorly (Fig. 8); posterior chamber degenerated, not filling body cavity, connected with anterior chamber by a short, slender tube.

##### Coloration.

In life, body coloration of *Triplophysarongduensis* sp. nov. yellowish and heavily pigmented (Fig. 9). After fixation in 10% formalin solution, the body color became slightly whitened (Fig. 7).

##### Secondary sex characteristics.

In one male holotype specimen (GZNU20230106001), microvillous microspinules were present on the anterior margin of the eye, outside of nostrils, upper lip, and outrostral barbel area.

##### Comparisons.

Comparative data of *Triplophysarongduensis* sp. nov. with *T.cehengensis* sp. nov. and the 34 recognized hypogean species within genus *Triplophysa* are given in Suppl. material 4. Based on the dorsal-fin origin anterior to the pelvic-fin origin and the unbranched pelvic-fin rays 2, new species can be placed within defined subclade B1. Thus, new species and morphological differences with subclade B1 and 15 undefined clade species were compared in detail.

*Triplophysarongduensis* sp. nov. differs from *T.cehengensis* sp. nov. by body pigmentation presence (vs absence), eye normal, diameter 7–15% of HL (vs eye reduced, diameter 2% of HL), dorsal fin distal margin emarginated (vs truncated), seven branched pelvic-fin rays (vs 8), tip of pectoral fin reaching to pelvic fin origin (vs not reaching to pelvic fin origin), tip of pelvic fin not reaching to anus (vs reaching to anus), and total vertebrae 43 (vs 39).

*Triplophysarongduensis* sp. nov. can be distinguished from *T.anshuiensis*, *T.langpingensis*, *T.qiubeiensis*, *T.aluensis*, *T.fengshanensis*, *T.gejiuensis*, *T.posterodorsalus*, and *T.shilinensis* by body pigmentation presence (vs absence) and eye normal, diameter 7–15% of HL (vs eye reduced or absence); from *T.huapingensis*, *T.flavicorpus*, *T.guizhouensis*, *T.longipectoralis*, *T.luochengensis*, and *T.yunnanensis* by body scaleless (vs body covered by sparse scales).

*Triplophysarongduensis* sp. nov. can be distinguished from *T.baotianensis*, *T.nanpanjiangensis*, *T.tianxingensis*, *T.xiangshuingensis*, and *T.xichouensis* by four unbranched dorsal-fin rays (vs 3) and two unbranched pectoral-fin rays (vs 1). *Triplophysarongduensis* sp. nov. can be futher distinguished from *T.baotianensis* by four unbranched dorsal -fin rays (vs 3), four three unbranched anal -fin rays (vs 2), 16 branched caudal-fin rays (vs 11–13), and ten branched pectoral-fin rays (vs 9); from *T.nanpanjiangensis*, *T.tianxingensis*, and *T.xichouensis* by three unbranched anal-fin rays (vs 2); and from *T.xiangshuingensis* by small interorbital width, 24–29% of HL (vs 31–43% of HL), nine branched dorsal-fin rays (vs 6), ten branched pectoral-fin rays (vs 8 or 9), and 16 branched caudal-fin rays (vs 14).

*Triplophysarongduensis* sp. nov. can be morphologically distinguished from its close relative *T.zhenfengensis* by dorsal fin distal margin emarginated (vs truncated), body scaleless (vs body covered by sparse scales), dorsal fin rays (iv, 9 vs iii, 7), ten branched pectoral-fin rays (vs 9), two unbranched pectoral-fin rays (vs 1), 16 branched caudal-fin rays (vs 14 or 15), tip of pectoral fin reaching to pelvic fin origin (vs not reaching to pelvic fin origin), and total vertebrae 43 (vs 40).

##### Ecology and distribution.

Currently, this new species *Triplophysarongduensis* sp. nov. has only been found in a cave in Rongbei Village, Rongdu Town, Ceheng County, Guizhou Province, China, at an elevation of 1228 m. The pool where the new species was discovered is more than 4.5 m long, 6 m wide, and 5 m deep, with a slow flow of water, and is located ~ 30 m vertically down from the cave entrance. Inside the cave, crabs (*Diyutamoncereum*, 25 individuals), *Sinocyclocheilus* sp., and *Oreolalaxrhodostigmatus* were found. Outside the caves, edible rape and rice are being grown. The caves in the habitat of this new species are the main source of drinking water for the local population.

##### Etymology.

The specific epithet *rongduensis* is in reference to the type locality of the new species: Rongdu Town, Ceheng County, Guizhou Province, China. We propose the common English name “Rongdu high-plateau loach” and the Chinese name “Rǒng Dù Gāo Yuán Qīu (冗渡高原鳅).”

#### ﻿ Triplophysa panzhouensis

Taxon classificationAnimaliaCypriniformesNemacheilidae

Yu, Luo, Lan, Xiao & Zhou sp. nov.

9A2698E3-C1A7-570D-8542-422FC6B4E55D

https://zoobank.org/9CAA27D2-579E-4A79-8229-C83E02518E4A

**Figure 10.**
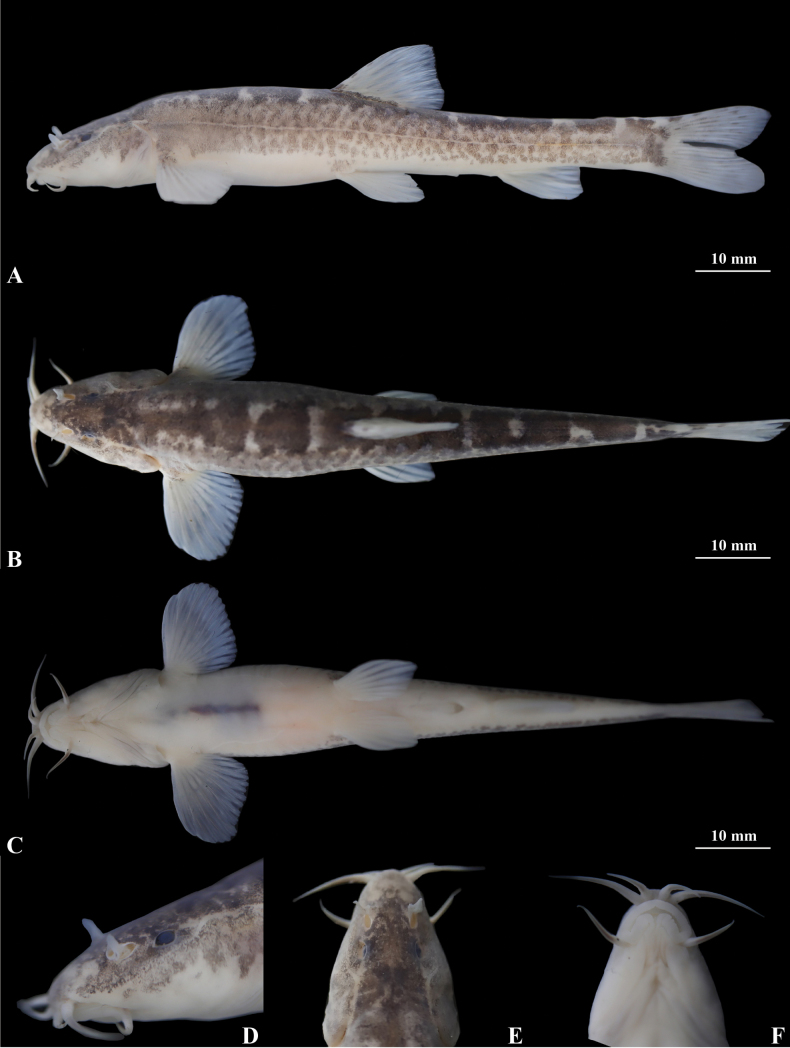
Morphological characteristics of holotype GZNU20230226002 of *Triplophysapanzhouensis* sp. nov. in preservative (10% formalin) **A** lateral view **B** dorsal view **C** ventral view **D** right side view of head **E** left side view of head, and **F** ventral view of head. Photographs by Tao Luo.

**Figure 11.**
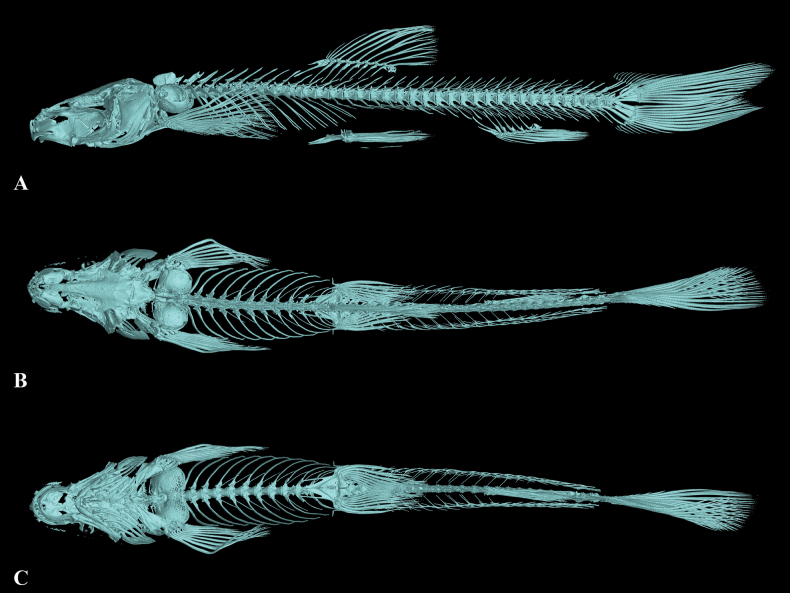
The three-dimensional reconstructed model of the skeleton of *Triplophysapanzhouensis* sp. nov (paratype GZNU20230223001, standard length 66.8 mm) **A** lateral view **B** dorsal view, and **C** ventral view.

**Figure 12.**
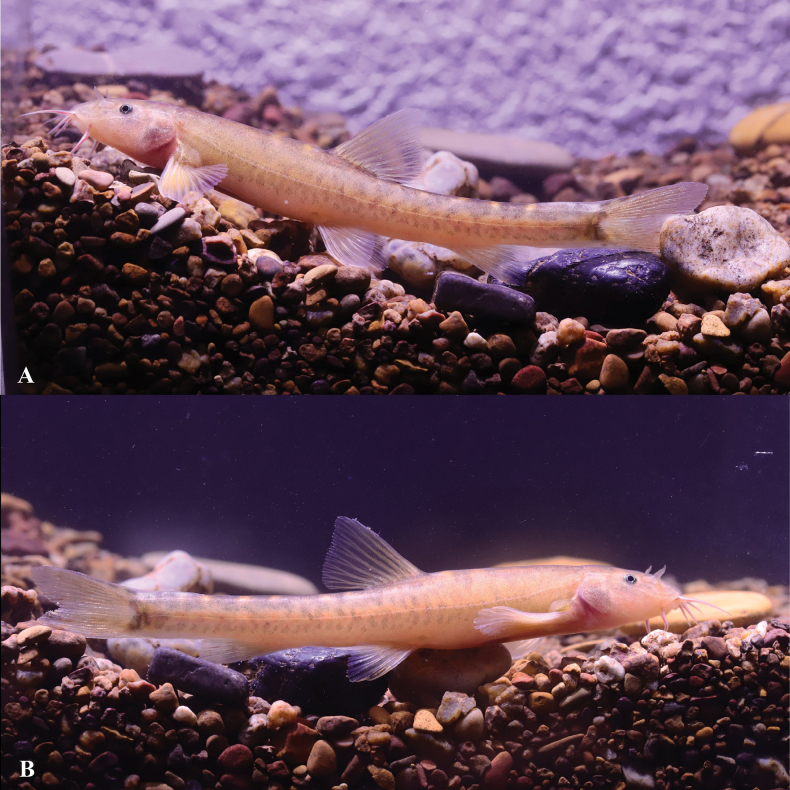
*Triplophysapanzhouensis* sp. nov. in life, paratype GZNU20230115001 **A** left side view **B** right side view.

Figs 10 , 11 , 12 , Suppl. materials 3 , 5

##### Type material.

***Holotype*.** GZNU20230226002 (Fig. 10), 106.3 mm total length (TL), 88.9 mm standard length (SL), collected by Tao Luo on February 15, 2023, in Hongguo Town, Panzhou City, Guizhou Province, China (25.6576°N, 104.4044°E; ca. 1852 m a.s.l.; Fig. 1).

***Paratypes*.** Eleven specimens from the same locality as the holotype: GZNU20230215025–215029, GZNU20230216042–216044, GZNU20230226001–226003, and GZNU20230105001 collected by Tao Luo, Xing-Liang Wang, Ya-Li Wang, Jing Yu, and Wei-Feng Wang on February 15, 2023.

##### Diagnosis.

*Triplophysapanzhouensis* sp. nov. can be distinguished from species of the hypogean group of its congeners by the following combination of characteristics: (1) body naked, scaleless, with skin pigmentation; (2) eyes normal, diameter 7–11% of HL, interorbital width 22–31% of HL; (3) dorsal fin distal margin truncated; (4) pelvic-fin tip not reaching to anus; (5) tip of pectoral-fin not reaching to pelvic fin origin; (6) anterior and posterior nostrils closely set, anterior nostril elongated to a barbel-like tip; (7) tip of outrostral barbel extending backward to anterior margin of the eye; (8) lateral line complete; (9) dorsal-fin rays iv-7–8, pectoral-fin rays i-11, pelvic-fin rays ii-7, anal-fin rays iii-5, 16 branched caudal-fin rays; and (10) total vertebrae 39.

##### Description.

Morphological data of the specimens of the *Triplophysapanzhouensis* sp. nov. are provided in Suppl. materials 3, 5. Body elongated and cylindrical, posterior portion gradually compressed from dorsal-fin to caudal-fin base, with deepest body depth anterior to dorsal-fin origin, deepest body depth 9–15% of snout length. Dorsal profile slightly convex from snout to dorsal-fin insertion, then straight from posterior portion of dorsal-fin origin to caudal-fin base. Ventral profile flat. Head slightly long, length 22–24% of SL, slightly depressed and flattened, width greater than depth (HW/HD = 1.3). Snout short, length 44–51% of HL, slightly pointed. Mouth inferior and curved, mouth corner situated below anterior nostril, upper and lower lips smooth, lips thick with shallow furrows, lower lip with a V-shaped median notch. Processus dentiformis absent. Upper and lower jaw arched. Three pairs of barbels are present: inrostral barbel short, length 20–27% of HL, backward extending to corner of the mouth; outrostral barbel long, length 39–53% of HL, backward extending to anterior margin of the eye. Maxillary barbel developed, length 28–41% of HL, tip of maxillary barbel reaching to anterior margin of operculum. Anterior and posterior nostrils closely set, length 0.3 mm. Anterior nostril tube long, with an elongated short barbel-like tip, elongated barbel-like tip of anterior nostril backwards not extending beyond the eyes. Eyes present, normal, diameter 7–11% of HL. Gill opening small, gill rakers not developed, nine inner gill rakers on first gill arch (*n* = 1).

Dorsal-fin rays iv-7–8, pectoral-fin rays i-11, pelvic-fin rays ii-7, anal-fin rays iii-5, 16 branched caudal-fin rays. Dorsal fin long, length 17–21% of HL, distally margin truncated, origin slightly anterior to pelvic-fin insertion, situated slightly posterior to midpoint between snout tip and caudal-fin base, first branched ray longest, shorter than head length, tip of dorsal fin not extending to vertical limit of anus. Pectoral fin moderately developed, length 15–20% of SL, tip of pectoral fin extends backwards not to the midpoint between origins of pectoral and pelvic fins, not reaching origin of pelvic fin. Pelvic fin length 13–17% of SL, vertically aligned with first branched ray of dorsal fin, tips of pelvic fin not reaching to anus, distance between tips of pelvic fin and anus 3.7× the eye diameter. Anal fin long, length 13–17% of SL, distal margin truncated, origin close to anus, spacing 4.6 mm, tips of anal fin not reaching caudal-fin base, distance between tips of anal fin and anus 3.1× the eye diameter. Caudal fin forked, upper lobe is equal in length to lower lobe, tips pointed, caudal peduncle length 12.0 mm, caudal peduncle depth 5.4 mm, without adipose crests along both dorsal and ventral sides. Total vertebrae 39 (*n* = 1).

Cephalic lateral line canals unclear. Lateral line complete and straight, exceeding tip of pectoral fin and reaching base of caudal fin. Two chambers of air bladder, anterior chamber dumbbell-shaped and membranous, open on both sides, slightly larger pores posteriorly (Fig. 11); posterior chamber degenerated, not filling body cavity, connected with anterior chamber by a short, slender tube.

##### Coloration.

In cave water bodies when alive, *Triplophysapanzhouensis* sp. nov. has light yellow skin with irregular light brown patches on the body (Fig. 12). After fixation in 10% formalin solution, the body color was light grey, and the light brown patches on the body became black and gray (Fig. 10).

##### Secondary sex characteristics.

No secondary sex characteristics was observed based on the present specimens of *Triplophysapanzhouensis* sp. nov.

##### Comparisons.

Comparative data of *Triplophysapanzhouensis* sp. nov. with *T.cehengensis* sp. nov., *T.rongduensis* sp. nov., and the 34 recognized hypogean species within genus *Triplophysa* are given in Suppl. material 4. Based on the dorsal-fin origin anterior to the pelvic-fin origin and the two unbranched pelvic-fin rays, new species can be placed within defined subclade B1. Thus, new species and morphological differences with subclade B1 and 15 undefined clade species were compared in detail.

*Triplophysapanzhouensis* sp. nov. differs from *T.cehengensis* sp. nov. by body pigmentation present (vs body pigmentation absent), eyes normal, diameter 7–11% of HL (vs eye reduced, diameter 2% of HL), dorsal fin distal margin truncated (vs emarginated), 7 branched pelvic-fin rays (vs 8), and tip of pelvic fin not reaching to anus (vs reaching to anus). *Triplophysapanzhouensis* sp. nov. differs from *T.rongduensis* sp. nov. by having nine branched dorsal-fin rays (vs 7 or 8), total vertebrae 39 (vs 43), and tip of outrostral barbel extending backward to anterior margin of the eye (vs extending backward to posterior margin of the eye).

*Triplophysapanzhouensis* sp. nov. can be distinguished from *T.langpingensis*, *T.qiubeiensis*, *T.anshuiensis*, *T.aluensis*, *T.fengshanensis*, *T.gejiuensis*, *T.posterodorsalus*, and *T.shilinensis* by body pigmentation presence (vs absence) and eye normal, diameter 7–11% HL(vs eye reduced or absence); from *T.zhenfengensis*, *T.huapingensis*, *T.flavicorpus*, *T.guizhouensis*, *T.longipectoralis*, *T.luochengensis*, and *T.yunnanensis* by body scaleless (vs body covered by sparse scales) and four unbranched dorsal-fin rays (vs 3).

*Triplophysapanzhouensis* sp. nov. can be distinguished from *T.baotianensis*, *T.flavicorpus*, *T.nanpanjiangensis*, *T.tianxingensis*, *T.xiangshuingensis*, and *T.xichouensis* by four unbranched dorsal-fin rays (vs 3) and two unbranched pectoral-fin rays (vs 1). *Triplophysapanzhouensis* sp. nov. can be futher distinguished from *T.baotianensis* by four unbranched dorsal-fin rays (vs 3), and 11 branched pectoral-fin rays (vs 9); from *T.flavicorpus* by interorbital width (vs 22.1–31.3% of HL vs 3.1–5.2% of HL), dorsal fin distal margin truncated (vs emarginated), and seven or eight branched dorsal-fin rays (vs 10); from *T.nanpanjiangensis*, *T.tianxingensis*, and *T.xichouensis* by three unbranched anal-fin rays (vs 2) and total vertebrae 39 (vs 40–42); and from *T.xiangshuingensis* by seven or eight branched dorsal-fin rays (vs 6), 11 branched pectoral-fin rays (vs 8 or 9), and 16 branched caudal-fin rays (vs 14).

##### Ecology and distribution.

*Triplophysapanzhouensis* sp. nov. is only known from the type locality, a vertical cave ~ 3 km from Hongguo Town, Panzhou city, Guizhou, China at an elevation of 2276 m. The cave was completely dark. Individuals of *T.panzhouensis* sp. nov. were located in a small pool ~ 25 m from the cave entrance. The pool was ~ 1.8 m wide and 80 cm deep, with a water temperature of ~ 16 °C at the time of collection and a water pH of 7.4. Within this cave, *T.panzhouensis* sp. nov. co-occurred with *Sinocyclocheiluslongicornus*, *Sinocyclocheilus* sp., and *Oreolalaxrhodostigmatus*. The arable land outside the cave was farmed to produce maize, wheat, and potatoes.

##### Etymology.

The specific epithet *panzhouensis* is in reference to the type locality of the new species: Hongguo Town, Panzhou City, Guizhou Province, China. We propose the common English name “Panzhou high-plateau loach” and the Chinese name “Pán Zhõu Gāo Yuán Qīu (盘州高原鳅).”

#### ﻿ Triplophysa anlongensis

Taxon classificationAnimaliaCypriniformesNemacheilidae

Lan, Song, Luo, Zhao, Xiao & Zhou sp. nov.

CBC6D4A0-7617-501F-BB37-5AE3B68004FB

https://zoobank.org/A9FF79CA-6BF6-4B85-A0C3-B0ABC248724E

**Figure 13.**
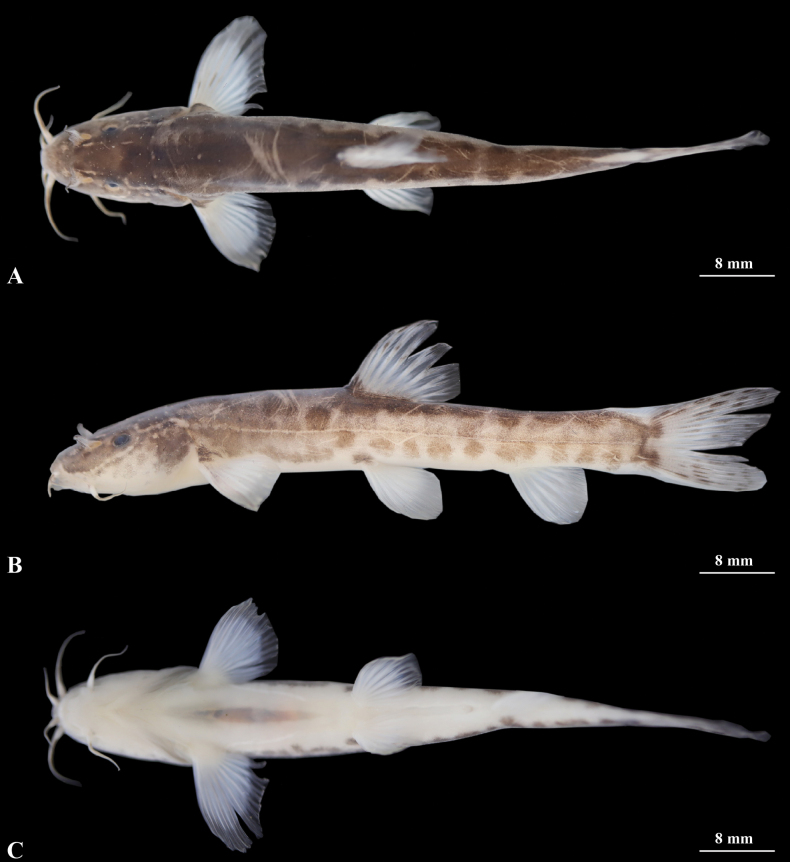
Morphological characteristics of holotype GZNU20230215021 of *Triplophysaanlongensis* sp. nov. in preservative (10% formalin) **A** lateral view **B** dorsal view **C** ventral view. Photographs by Tao Luo.

**Figure 14.**
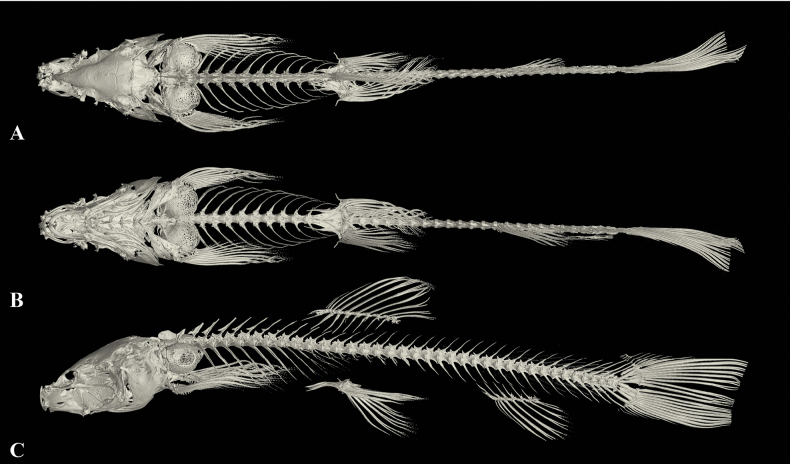
The three-dimensional reconstructed model of the skeleton of *Triplophysaanlongensis* sp. nov (paratype GZNU20230215022, standard length 51.1 mm) **A** dorsal view **B** ventral view, and **C** lateral view.

**Figure 15.**
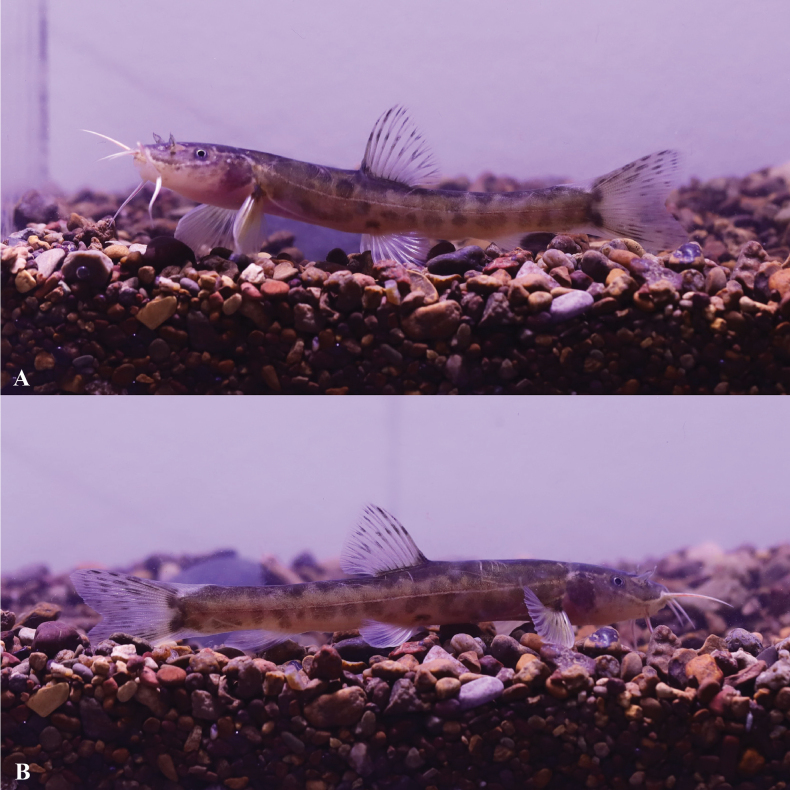
*Triplophysaanlongensis* sp. nov. in life, paratype GZNU20230215022 **A** left side view **B** right side view.

Figs 13 , 14 , 15 , Suppl. materials 3 , 5

##### Type material.

***Holotype*.** GZNU20230215021 (Fig. 13), 78.4 mm total length (TL), 65.45 mm standard length (SL), collected by Tao Luo on February 15, 2023, in NaNao Village, Xinglong Town, Anlong County, Guizhou Province, China (25.07096446°N, 105.59143424°E; ca. 1450 m a.s.l.; Fig. 1).

***Paratypes*.** Six specimens from the same locality as the holotype: GZNU20230215021–215024, GZNU20230226004, and GZNU20230226005, collected by Tao Luo, Wei-Feng Wang, Jing Yu, Chang-Ting Lan, and Xin-Rui Zhao on February 15, 2023.

##### Diagnosis.

*Triplophysaanlongensis* sp. nov. can be distinguished from all other congeners by the following combination of characteristics: (1) body naked, scaleless, without skin pigmentation; (2) eyes normal, diameter 5–9% of HL, interorbital width 32–36% of HL; (3) dorsal fin distal margin truncated; (4) pelvic-fin tip not reaching to anus; (5) tip of pectoral fin not reaching to pelvic fin origin; (6) anterior and posterior nostrils closely set, anterior nostril elongated to a barbel-like tip; (7) tip of outrostral barbe backward extending to middle of the eye; (8) lateral line complete; (9) dorsal-fin rays iii-8, pectoral-fin rays i-11, pelvic-fin rays ii-8, anal-fin rays iii-5, and 16 branched caudal-fin rays; and (10) total vertebrae 41.

##### Description.

Morphological data of the six specimens of the *Triplophysaanlongensis* sp. nov. are provided in Suppl. materials 3, 5. Body elongated and cylindrical, posterior portion gradually compressed from dorsal fin to caudal-fin base, with deepest body depth anterior to dorsal-fin origin, deepest body depth 13–16% of SL. Dorsal profile slightly convex from snout to dorsal-fin insertion, then straight from posterior portion of dorsal-fin origin to caudal-fin base. Ventral profile flat. Head slightly long, length 24–26% of SL, slightly depressed and flattened, width greater than depth (HW/HD = 1.2). Snout short, length 44–48% of HL, slightly blunt. Mouth inferior and curved, mouth corner situated below anterior nostril, upper and lower lips smooth, lips thick with shallow furrows, lower lip with a V-shaped median notch. Processus dentiformis absent. Upper and lower jaw arched. Three pairs of barbels are present: inrostral barbel short, length 23–31% of HL, backward extending to corner of the mouth; outrostral barbel long, 36–53% of HL, backward extending to middle of the eye. Maxillary barbel developed, length 31–48% of HL, tip of maxillary barbel reaching to posterior margin of operculum. Anterior and posterior nostrils closely set, length 0.4 mm. Anterior nostril tube long, with an elongated short barbel-like tip, elongated barbel-like tip of posterior nostril extending backwards reaching to anterior margin of the eye. Eyes present, normal, diameter 1.0 mm, 5–9% of HL. Gill opening small, gill rakers not developed, nine inner gill rakers on the first gill arch (*n* = 1).

Dorsal-fin rays iii-8, pectoral-fin rays i-11, pelvic-fin rays ii-8, anal-fin rays iii-5, and 16 branched caudal-fin rays. Dorsal fin long, length 18–22% of HL, distally margin truncated, origin slight anterior to pelvic-fin insertion, situated slightly posterior to midpoint between snout tip and caudal-fin base, first branched ray longest, shorter than head length, tip of dorsal fin not extending to vertical of anus. Pectoral fin moderately developed, length 16–22% of SL, tip of pectoral fin extends backwards not reaching the midpoint between the pectoral and pelvic fin origins. Pelvic fin length 14–16% of SL, vertically aligned with third branched ray of dorsal fin, tips of pelvic fin not reaching to anus, distance between tips of pelvic fin and anus 1.5× the eye diameter. Anal fin long, length 13–17% of SL, distally margin truncated, origin close to anus, spacing 1.3 mm, tips of anal fin backwards not reaching caudal-fin base, distance between tips of anal fin and anus 1.8× eye diameter. Caudal fin forked, upper lobe equal in length to lower lobe, tips pointed, caudal peduncle length 7.7 mm, caudal peduncle depth 4.5 mm, without adipose crests along both dorsal and ventral sides. Total vertebrae 41 (*n* = 1).

Cephalic lateral line canals unclear. Lateral line complete and straight, exceeding tip of pectoral fin and reaching base of caudal fin. Two chambers of air bladder, anterior chamber dumbbell-shaped and membranous, open on both sides, slightly closed posteriorly (Fig. 14); posterior chamber developed, slight filling body cavity, connected with anterior chamber by a long, slender tube.

##### Coloration.

In cave water bodies when alive, *Triplophysaanlongensis* sp. nov. has the ground color of body light yellow, slightly lighter ventrally. Dorsal and lateral parts of body and head gray and black. Dorsal, pectoral, pelvic, and anal fin rays brown, fin membrane hyaline. Blotches present on dorsal, pectoral, and caudal fins. Pelvic and anal fins without blotches (Fig. 15). After fixation in 10% formalin solution, the body color was light grey, and the light brown patches on the body became black and gray (Fig. 13).

##### Secondary sex characteristics.

No secondary sex characteristics was observed based on the present specimens of *Triplophysaanlongensis* sp. nov.

##### Comparisons.

Comparative data of *Triplophysaanlongensis* sp. nov. with *T.cehengensis* sp. nov., *T.rongduensis* sp. nov., *Triplophysapanzhouensis* sp. nov. and the 34 recognized hypogean species within genus *Triplophysa* are given in Suppl. material 4. Based on the dorsal-fin origin anterior to the pelvic-fin origin and the unbranched pelvic-fin rays 2, new species can be placed within defined subclade B1. Thus, new species and morphological differences with subclade B1 and 15 undefined clade species were compared in detail.

*Triplophysaanlongensis* sp. nov. differs from *T.cehengensis* sp. nov. By body pigmentation present (vs body pigmentation absent), eyes normal, diameter 5–9% of HL (vs eyes reduced, diameter 2% of HL), dorsal-fin rays (iii, 8 vs iv, 9), and tip of pelvic fin not reaching to anus (vs reaching to anus); from *T.rongduensis* sp. nov. by seven branched pelvic-fin rays (vs 6) and large interorbital width, 32–36% of HL (vs 24–29 of HL); and from *Triplophysapanzhouensis* sp. nov. by total vertebrae 41 (vs 39), dorsal fin rays (iii, 8 vs iv, 7–8), and eight branched pelvic fin rays (vs 7).

*Triplophysaanlongensis* sp. nov. can be distinguished from *T.langpingensis*, *T.qiubeiensis*, *T.anshuiensis*, *T.fengshanensis*, *T.gejiuensis*, *T.posterodorsalus*, *T.shilinensis*, and *T.xichouensis* body pigmentation presence (vs absence). *Triplophysaanlongensis* sp. nov. can be distinguished from *T.flavicorpus*, *T.longipectoralis*, *T.xiangshuingensis*, and *T.yunnanensis* by dorsal fin distal margin truncated (vs emarginated). *Triplophysaanlongensis* sp. nov. differs from *T.zhenfengensis*, *T.huapingensis*, *T.guizhouensis*, and *T.luochengensis* by body scaleless (vs body covered by sparse scales). *Triplophysaanlongensis* sp. nov. can be further distinguished from *T.langpingensis*, *T.qiubeiensis*, *T.anshuiensis*, *T.flavicorpus*, and *T.longipectoralis* by tip of pelvic fin not reaching to anus (vs reaching to anus).

*Triplophysaanlongensis* sp. nov. differs from *T.baotianensis* by slight small eyes, diameter 5–9% of HL (vs 9–13% of HL), large interorbital width, 32–36% of HL (vs 3–5% of HL), eight branched dorsal fin rays (vs 6 or 7), three unbranched anal-fin rays (vs 2), 11 branched pectoral-fin rays (vs 9), pelvic-fin rays (ii, 8 vs i, 6 or 7), and six branched caudal-fin rays (vs 11–13); from *T.tianxingensis* by anterior nostril elongated to a short barbel-like tip (vs not elongated to a short barbel-like tip), three unbranched anal-fin rays (vs 2), 11 branched pectoral-fin rays (vs 9), pelvic-fin rays (ii, 8 vs i, 5–7), and total vertebrae 41 (vs 42); and from *T.xichouensis* by anal-fin rays (iii, 5 vs ii, 6), 11 branched pectoral-fin rays (vs 9 or 10), pelvic-fin rays (ii, 8 vs i, 5 or 6), tip of pelvic fin not reaching to anus (vs reaching to anus), and total vertebrae 41 (vs 40).

##### Ecology and distribution.

*Triplophysaanlongensis* sp. nov. is only known from the type locality, a vertical cave ~ 3 km from NaNao Village, Xinglong Town, Anlong County, Guizhou Province, China at an elevation of 1387 m. There is no surface stream outside the cave. The cave is approximately 50 m long and has a small volume of water during dry periods, although it is a source of domestic water for the local population. Within this cave, *Triplophysaanlongensis* sp. nov. co-occurred with fish (*Balitoraanlongensis* and *Misgurnusanguillicaudatus*), frogs (*Odorrana* sp.), the red-eared slider (*Trachemysscripta*), and crabs (*Diyutamoncereum*). Outside the cave, the arable land was farmed to produce maize, wheat, and potatoes.

##### Etymology.

The specific epithet *anlongensis* is in reference to the type locality of the new species: NaNao Village, Xinglong Town, Anlong County, Guizhou Province, China. We propose the common English name “Anlong high-plateau loach” and the Chinese name “ān lóng Gāo Yuán Qīu (安龙高原鳅).”

## ﻿Discussion

In this work, we have described four new species named *T.cehengensis* sp. nov., *T.rongduensis* sp. nov., *T.panzhouensis* sp. nov., and *T.anlongensis* sp. nov. based on morphological comparisons and mitochondrial DNA sequence differences (Fig. 2, Table 3 and Suppl. material 4). The description of these four new species increases the number of species in the hypogean group of the genus *Triplophysa* from 35 to 39, with the number of known species from Guizhou increasing to 12: *T.baotianensis*, *T.guizhouensis*, *T.longliensis*, *T.nasobarbatula*, *T.qingzhenensis*, *T.wudangensis*, *T.sanduensis*, *T.zhenfengensis* (Table 1), and the four new species described in this study. Notably, *Triplophysa* species were previously discovered in Guizhou in scattered areas in the west, center, and south (Fig. 1). The existence of large gaps in records between these areas, especially in the Beipanjiang, Nanpanjiang, and Hongshui rivers of the Pearl River system and the upper reaches of the Wujiang River in the Yangtze River system (Fig. 1), implies that there should be further undescribed species or extant species ranges in these areas. In other words, the species diversity of *Triplophysa* in Guizhou Province may be underestimated and thus needs to be fully and systematically surveyed. However, based on our current data, many of the records are in caves at vertical depths of several tens of meters, thus requiring the effort and cooperation of multiple parties, including professional taxonomic researchers and hobbyists exploring caves. Example species that have been described in this way include *T.qini* and *T.rosa* (Chen and Yang 2005; Deng et al. 2022).

Cave-dwelling species of the genus *Triplophysa* distributed in the karst region of southwest China, including Guangxi, Guizhou, Yunnan, Chongqing, and Hunan provinces (Fig. 1) would be suitable candidates for exploring the evolution of the Yangtze and Pearl River systems. In the phylogenetic tree reconstructed based on mitochondrial Cyt *b*, species from the Yangtze and Pearl River drainages do not strictly cluster together (Fig. 2). *Tryplophysawulongensis* from Chongqing became the basal Clade A of the hypogean group of the genus *Triplophysa*, which, based on our own field surveys, also occurs in Dafang County in the upper reaches of the Wujiang River. Clade B1 from the Pearl River system and Clade B2 comprise mixtures of species from the Pearl and Yangtze River systems, and clades B1 and B2 have *T.qiubeiensis* and *T.xuanweiensis* from the upper Pearl River in Yunnan as basal clades. This information suggests that the ancestors of the hypogean group of *Triplophysa* may have come from the ancient Yangtze River, and that the Yangtze and Pearl River systems were historically interconnected (Yan 2017). To test this hypothesis, full surveys and the use of additional genetic data such as complete genomes from fishes in the karst region of southwest China are essential.

The Yangtze River system (seven species) has a low number of species with highly specialized phenotypes (Liu et al. 2022) such as eye and body pigmentation (absent or reduced) compared to the Pearl River system (28 species) (Fig. 2, Table 1), a pattern that may be related to karst development. Within Clade B, morphologically specialized species account for 11.1% and 69.3% of the species, with the Yangtze River system dominating (Fig. 2). Combining the phylogeny and species numbers suggests that species from the Yangtze River system may have occupied the caves for a longer period of time (Yan 2017), whereas the fishes from the Pearl River system have recently undergone rapid dispersal followed by isolated differentiation to form new species (Yan 2017; Wen et al. 2022), and this has been influenced by the uplift of the Qinghai-Tibet Plateau and heavy rainfall (Deng et al. 2020; Vornlocher et al. 2021; Wu et al. 2022).

## Supplementary Material

XML Treatment for Triplophysa cehengensis

XML Treatment for Triplophysa rongduensis

XML Treatment for Triplophysa panzhouensis

XML Treatment for Triplophysa anlongensis

## ﻿Additional information

### Author ORCIDs

Tao Luo https://orcid.org/0000-0003-4186-1192

Ming-Le Mao https://orcid.org/0000-0001-9764-4731

Chang-Ting Lan https://orcid.org/0009-0007-2381-3601

Ling-Xing Song https://orcid.org/0009-0007-2897-1852

Xin-Rui Zhao https://orcid.org/0000-0002-9125-6276

Jing Yu https://orcid.org/0009-0004-3629-3826

Xing-Liang Wang https://orcid.org/0009-0003-0654-8640

Ning Xiao https://orcid.org/0000-0002-7240-6726

Jia-Jun Zhou https://orcid.org/0000-0003-1038-1540

Jiang Zhou https://orcid.org/0000-0003-1560-8759

### Data availability

All of the data that support the findings of this study are available in the main text or Supplementary Information.

## ﻿Appendix 1

**Specimens examined in this work**

***Triplophysabaotianensis*** (*n* = 5): China: Guizhou: Panzhou City: Baotian Town (type locality): GZNU 20180421001–421005.

***Triplophysaerythraea*** (*n* = 2): China: Hunan: Huayuan County: Dalong Cave (type locality): GZNU20230216052 and GZNU20230216053.

***Triplophysanasobarbatula*** (*n* = 8): China: Guizhou: Libo County: Dongtang Township (type locality): GZNU 20170731003–0731010.

***Triplophysaguizhouensis*** (*n* = 6): China: Guizhou: Huisui County: Baijin Town (type locality): GZNU 20220715001–0715006.

***Triplophysazhenfengensis*** (*n* = 5): China: Guizhou: Xingren City: Xinlongchang Town: GZNU20180419002, GZNU20190521001–052104.

***Triplophysalangpingensis*** (*n* = 2): China: Guangxi: Leye County: Huaping Township (type locality): GZNU 20221209001–1209002.

***Triplophysamacrocephala*** (*n* = 8): China: Guangxi: Nandan County: Lihu Township (type locality): GZNU 20221209003–1209010.

***Triplophysatianeensis*** (*n* = 7): China: Guangxi: Tian’e County: Bala Township (type locality): GZNU 20221209011–1209017.

***Triplophysahuapingensis*** (*n* = 7): China: Guangxi: Leye County: Huaping Township (type locality): GZNU 20221209012–1209019.

***Triplophysanandanensis*** (*n* = 2): China: Guangxi: Nandan County: Liuzhai Town (type locality): GZNU 20221209020–1209021.

***Triplophysaqiubeiensis*** (*n* = 2): China: Yunan: Qiubei County: Nijiao Town (type locality): GZNU 20221209022–1209023.

***Triplophysaqingzhenensis*** (*n* = 8): China: Guizhou: Guiyang City: Qingzhen County (type locality): GZNU20220830001–0830008.

***Triplophysaqini*** (*n* = 5): China: Chongqing: Wulong District: Fengdu County: Dudu Village (type locality): GZNU20230216045–0216045.

***Triplophysawudangensis*** (*n* = 4): China: Guizhou: Guiyang City: Wudang District (type locality): IHB 201908090001–090004.

***Triplophysarosa*** (*n* = 9): China: Chongqing: Wulong County: Huolu Town: GZNU20230216070–0216078.

## Supplementary materials

Supplementary material 1 Boxplots of morphometrics based on 35 morphometric measurements distinguishing *T.anlongensis* sp. nov., *T.cehengensis* sp. nov., *T.panzhouensis* sp. nov., *T.rongduensis* sp. nov., *T.baotianensis*, and *T.zhenfengensis* Data type png https://binary.pensoft.net/file/943702This dataset is made available under the Open Database License (http://opendatacommons.org/licenses/odbl/1.0/). The Open Database License (ODbL) is a license agreement intended to allow users to freely share, modify, and use this Dataset while maintaining this same freedom for others, provided that the original source and author(s) are credited.Tao Luo, Ming-Le Mao, Chang-Ting Lan, Ling-Xing Song, Xin-Rui Zhao, Jing Yu, Xing-Liang Wang, Ning Xiao, Jia-Jun Zhou, Jiang Zhou

Supplementary material 2 GPS information on the geographical distribution of 39 hypogean species of the genus *Triplophysa* Data type xlsx https://binary.pensoft.net/file/943703This dataset is made available under the Open Database License (http://opendatacommons.org/licenses/odbl/1.0/). The Open Database License (ODbL) is a license agreement intended to allow users to freely share, modify, and use this Dataset while maintaining this same freedom for others, provided that the original source and author(s) are credited.Tao Luo, Ming-Le Mao, Chang-Ting Lan, Ling-Xing Song, Xin-Rui Zhao, Jing Yu, Xing-Liang Wang, Ning Xiao, Jia-Jun Zhou, Jiang Zhou

Supplementary material 3 Morphological characters and measurement data Data type xlsx Explanation note Morphological characters and measurement data of the new species *Triplophysacehengensis* sp. nov., *Triplophysarongduensis* sp. nov., *Triplophysapanzhouensis* sp. nov., *Triplophysaanlongensis* sp. nov., *T.zhenfengensis*, and *T.baotianensis*. *Designates the holotype. https://binary.pensoft.net/file/943704This dataset is made available under the Open Database License (http://opendatacommons.org/licenses/odbl/1.0/). The Open Database License (ODbL) is a license agreement intended to allow users to freely share, modify, and use this Dataset while maintaining this same freedom for others, provided that the original source and author(s) are credited.Tao Luo, Ming-Le Mao, Chang-Ting Lan, Ling-Xing Song, Xin-Rui Zhao, Jing Yu, Xing-Liang Wang, Ning Xiao, Jia-Jun Zhou, Jiang Zhou

Supplementary material 4 Comparison of the diagnostic features of the four new species described here with those selected for the 35 recognized hypogean species of the genus *Triplophysa* Data type xlsx Explanation note Modified from Wu et al. (2018) and Liu et al. (2022). “–” indicates uncertainty. https://binary.pensoft.net/file/943705This dataset is made available under the Open Database License (http://opendatacommons.org/licenses/odbl/1.0/). The Open Database License (ODbL) is a license agreement intended to allow users to freely share, modify, and use this Dataset while maintaining this same freedom for others, provided that the original source and author(s) are credited.Tao Luo, Ming-Le Mao, Chang-Ting Lan, Ling-Xing Song, Xin-Rui Zhao, Jing Yu, Xing-Liang Wang, Ning Xiao, Jia-Jun Zhou, Jiang Zhou

Supplementary material 5 Morphological comparison of *T.cehengensis* sp. nov. (*TC*), *T.rongduensis* sp. nov. (*TR*), *T.panzhouensis* sp. nov. (*TP*), *T.anlongensis* sp. nov. (*TA*), *T.baotianensis* (*TB*), and *T.zhenfengensis* (*TZ*) Data type docx Explanation note All units in mm. *P*-values are at the 95% significance level. https://binary.pensoft.net/file/943706This dataset is made available under the Open Database License (http://opendatacommons.org/licenses/odbl/1.0/). The Open Database License (ODbL) is a license agreement intended to allow users to freely share, modify, and use this Dataset while maintaining this same freedom for others, provided that the original source and author(s) are credited.Tao Luo, Ming-Le Mao, Chang-Ting Lan, Ling-Xing Song, Xin-Rui Zhao, Jing Yu, Xing-Liang Wang, Ning Xiao, Jia-Jun Zhou, Jiang Zhou

Supplementary material 6 Results and percentage of variance explained by principal component and discriminant function analyses Data type xlsx https://binary.pensoft.net/file/943707This dataset is made available under the Open Database License (http://opendatacommons.org/licenses/odbl/1.0/). The Open Database License (ODbL) is a license agreement intended to allow users to freely share, modify, and use this Dataset while maintaining this same freedom for others, provided that the original source and author(s) are credited.Tao Luo, Ming-Le Mao, Chang-Ting Lan, Ling-Xing Song, Xin-Rui Zhao, Jing Yu, Xing-Liang Wang, Ning Xiao, Jia-Jun Zhou, Jiang Zhou
