# Supplementary figures and images for: ﻿Four new hypogean species of the genus Triplophysa (Osteichthyes, Cypriniformes, Nemacheilidae) from Guizhou Province, Southwest China, based on molecular and morphological data
Source: Zookeys. 2023 Nov 28;1185:43–81. doi: 10.3897/zookeys.1185.105499 (PMC10698870; doi:10.3897/zookeys.1185.105499)

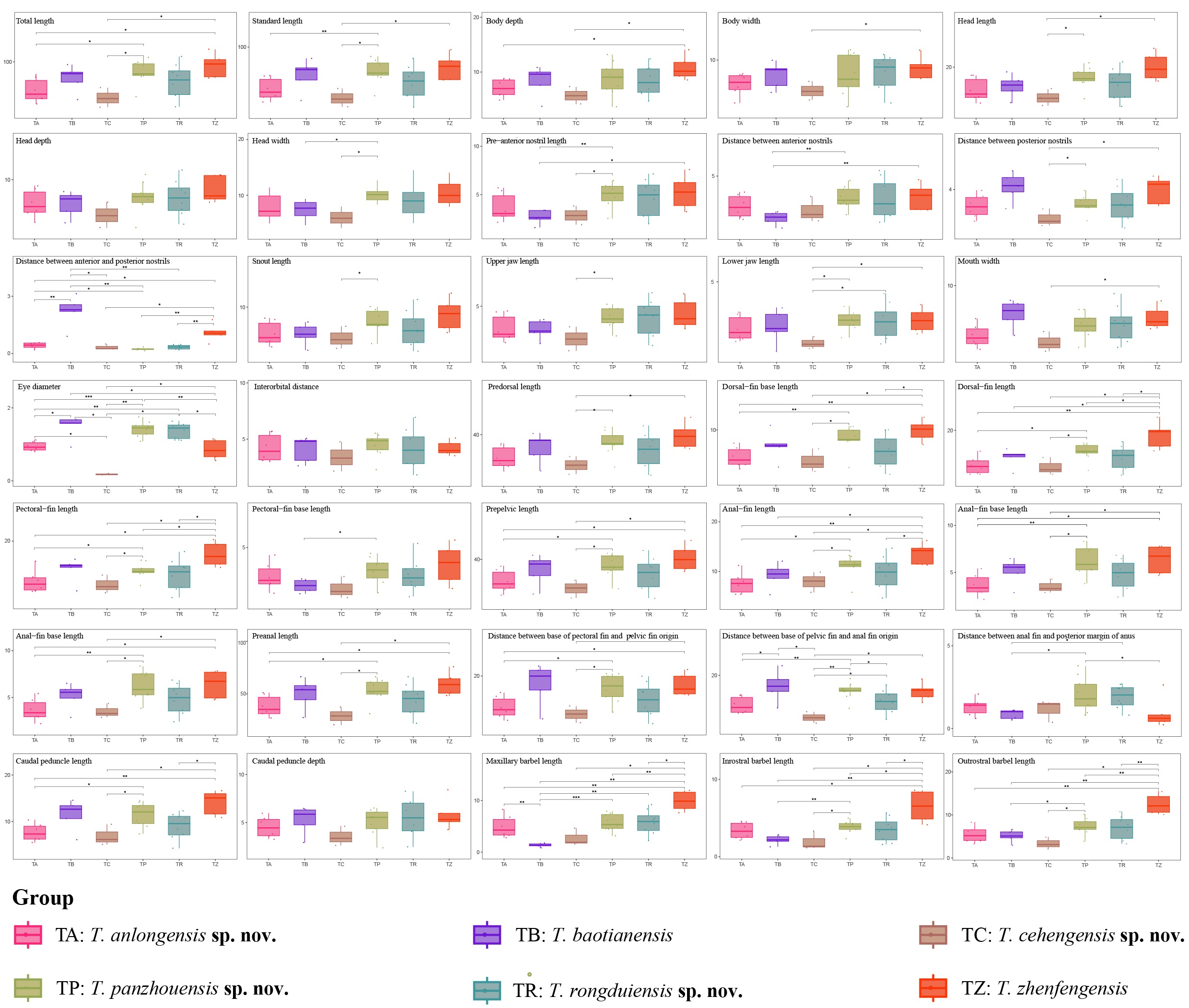

Supplement: Supplementary material 1 — Boxplots of morphometrics based on 35 morphometric measurements distinguishing T.anlongensis sp. nov., T.cehengensis sp. nov., T.panzhouensis sp. nov., T.rongduensis sp. nov., T.baotianensis, and T.zhenfengensis [file zookeys-1185-043_article-105499__-s001.png]
