# Supplementary material for: ﻿Four new hypogean species of the genus Triplophysa (Osteichthyes, Cypriniformes, Nemacheilidae) from Guizhou Province, Southwest China, based on molecular and morphological data
Source: Zookeys. 2023 Nov 28;1185:43–81. doi: 10.3897/zookeys.1185.105499 (PMC10698870; doi:10.3897/zookeys.1185.105499)
Supplement: Supplementary material 5 — Morphological comparison of T.cehengensis sp. nov. (TC), T.rongduensis sp. nov. (TR), T.panzhouensis sp. nov. (TP), T.anlongensis sp. nov. (TA), T.baotianensis (TB), and T.zhenfengensis (TZ) [file zookeys-1185-043_article-105499__-s005.docx]

**Table S4.** Morphological comparison of *T. cehengensis* **sp. nov.** (*TC*), *T. rongduiensis* **sp. nov.** (*TR*), *T. panzhouensis* **sp. nov.** (*TP*), *T. anlongensis* sp. nov. (*TA*), *T. baotianensis* (*TB*), and *T. zhenfengensis* (*TZ*). All units in mm. *P*-values are at the 95% significance level.

|  | *T. cehengensis* sp. nov.  （*N*=3) | | *T. rongduiensis* sp. nov.  （*N*=7) | | *T. panzhouensis* sp. nov.  （*N*=8) | | *T. anlongensis* sp. nov.  （*N*=6) | | *T. baotianensis*  （*N*=5) | | *T. zhenfengensis*  （N=5) | | *TC* vs. *TR* |
| --- | --- | --- | --- | --- | --- | --- | --- | --- | --- | --- | --- | --- | --- |
|  | Range | Mean ± SD | Range | Mean ± SD | Range | Mean ± SD | Range | Mean ± SD | Range | Mean ± SD | Range | Mean ± SD |  |
| Total length | 36.6–63.8 | 49.7 ± 13.6 | 53.3–107.7 | 77.8 ± 20.3 | 79.4–106.3 | 90.4 ± 10.3 | 41.1–81.9 | 59.9 ± 16.9 | 47.0–96.2 | 76.8 ± 18.8 | 77.6–117.4 | 94.9 ± 16.8 | 0.133 |
| Standard length | 29.5–51.2 | 39.8 ± 10.9 | 42.4–86.6 | 62.4 ± 16.5 | 64.3–88.9 | 75.2 ± 9.2 | 35.1–66.4 | 50.2 ± 13.2 | 37.1–86.5 | 66.7 ± 18.8 | 61.5–96.5 | 76.1 ± 15.0 | 0.194 |
| Body depth | 4.2–7.3 | 5.7 ± 1.6 | 6.2–12.4 | 8.9 ± 2.4 | 6.5–13.2 | 9.4 ± 2.4 | 5.0–8.9 | 7.1 ± 1.7 | 3.8–10.9 | 8.4 ± 2.8 | 8.6–14.1 | 10.8 ± 2.2 | 0.194 |
| Body width | 3.2–6.4 | 4.7 ± 1.6 | 5.4–11.0 | 8.6 ± 2.2 | 4.3–11.8 | 8.1 ± 2.9 | 2.7–7.6 | 5.8 ± 1.9 | 4.4–9.9 | 7.4 ± 2.3 | 6.8–11.7 | 8.7 ± 2.0 | 0.194 |
| Head length | 7.8–13.0 | 10.3 ± 2.6 | 10.2–21.6 | 15.6 ± 4.3 | 15.1–21.2 | 17.6 ± 2.1 | 8.6–17.4 | 12.8 ± 3.7 | 8.7–18.3 | 13.9 ± 3.6 | 15.4–25.8 | 20.0 ± 4.4 | 0.194 |
| Head depth | 3.6–7.0 | 5.3 ± 1.7 | 5.5–11.3 | 8.0 ± 2.1 | 6.7–10.7 | 8.2 ± 1.4 | 4.3–9.2 | 6.8 ± 1.9 | 4.3–8.4 | 6.8 ± 1.7 | 7.0–10.6 | 8.7 ± 1.8 | 0.194 |
| Head width | 4.1–8.0 | 6.0 ± 2.0 | 6.6–14.5 | 9.8 ± 2.9 | 8.3–13.8 | 10.7 ± 1.8 | 5.0–11.4 | 7.9 ± 2.6 | 4.6–9.4 | 7.3 ± 1.9 | 7.9–14.0 | 10.5 ± 2.5 | 0.133 |
| Pre-anterior nostril length | 1.9–3.8 | 2.8 ± 1.0 | 2.8–7.2 | 5.0 ± 1.7 | 4.1–6.5 | 5.3 ± 0.8 | 2.2–5.7 | 3.7 ± 1.5 | 1.6–3.4 | 2.7 ± 0.7 | 3.2–7.6 | 5.3 ± 1.8 | 0.194 |
| Distance between anterior nostrils | 1.6–3.5 | 2.4 ± 1.0 | 2.0–5.4 | 3.5 ± 1.4 | 2.8–4.7 | 3.5 ± 0.7 | 1.7–3.8 | 2.7 ± 0.9 | 1.0–2.3 | 1.8 ± 0.5 | 2.4–4.7 | 3.4 ± 1.0 | 0.497 |
| Distance between posterior nostrils | 1.6–2.7 | 2.0 ± 0.6 | 2.1–4.7 | 3.3 ± 1.0 | 2.8–4.0 | 3.2 ± 0.4 | 1.9–4.0 | 2.9 ± 0.8 | 2.7–5.3 | 4.2 ± 1.0 | 3.0–5.0 | 4.0 ± 0.9 | 0.194 |
| Distance between anterior and posterior nostrils | 0.2–0.5 | 0.3 ± 0.2 | 0.2–0.5 | 0.4 ± 0.1 | 0.2–0.3 | 0.3 ± 0.1 | 0.2–0.6 | 0.4 ± 0.2 | 0.9–3.1 | 2.2 ± 0.8 | 0.5–1.8 | 1.1 ± 0.5 | 0.776 |
| Snout length | 3.7–7.1 | 5.3 ± 1.7 | 4.3–11.2 | 7.2 ± 2.5 | 7.2–10.0 | 8.3 ± 1.2 | 3.9–8.3 | 5.9 ± 1.9 | 3.4–7.7 | 5.9 ± 1.6 | 6.1–12.1 | 8.9 ± 2.4 | 0.630 |
| Upper jaw length | 1.3–3.3 | 2.3 ± 1.0 | 2.5–6.1 | 4.3 ± 1.3 | 3.5–5.2 | 4.2 ± 0.7 | 2.0–4.6 | 3.1 ± 1.1 | 1.9–3.9 | 3.0 ± 0.8 | 3.1–6.0 | 4.4 ± 1.2 | 0.133 |
| Lower jaw length | 1.2–1.9 | 1.5 ± 0.4 | 1.8–4.3 | 2.9 ± 0.9 | 2.4–3.6 | 2.9 ± 0.4 | 1.6–3.3 | 2.4 ± 0.7 | 1.1–3.5 | 2.4 ± 0.9 | 2.1–3.7 | 2.8 ± 0.7 | 0.048 |
| Mouth width | 2.1–4.5 | 3.2 ± 1.2 | 3.3–9.0 | 5.8 ± 2.0 | 4.1–7.1 | 5.5 ± 1.2 | 2.3–6.0 | 3.9 ± 1.4 | 4.0–8.2 | 6.6 ± 1.7 | 4.8–8.1 | 6.1 ± 1.4 | 0.133 |
| Eye diameter | 0.2–0.2 | 0.2 ± 0.0 | 1.1–1.6 | 1.4 ± 0.2 | 1.2–1.8 | 1.5 ± 0.2 | 0.8–1.1 | 1.0 ± 0.1 | 0.9–1.7 | 1.5 ± 0.3 | 0.6–1.2 | 0.9 ± 0.3 | 0.012 |
| Interorbital distance | 2.1–4.8 | 3.4 ± 1.4 | 2.7–6.9 | 4.5 ± 1.6 | 3.8–5.6 | 4.8 ± 0.6 | 3.0–5.7 | 4.2 ± 1.2 | 2.6–5.1 | 4.1 ± 1.2 | 3.5–5.1 | 4.2 ± 0.6 | 0.630 |
| Predorsal length | 15.4–26.5 | 21.0 ± 5.6 | 20.9–45.7 | 32.4 ± 9.1 | 30.2–44.9 | 37.2 ± 5.0 | 17.7–34.6 | 25.7 ± 7.1 | 17.6–41.0 | 31.9 ± 9.4 | 31.4–51.1 | 39.5 ± 8.1 | 0.279 |
| Dorsal-fin base length | 4.0–7.3 | 5.4 ± 1.7 | 4.7–10.1 | 7.4 ± 2.2 | 8.4–10.6 | 9.3 ± 0.9 | 4.3–8.1 | 6.0 ± 1.5 | 4.6–10.7 | 7.7 ± 2.2 | 7.9–11.9 | 9.9 ± 1.6 | 0.497 |
| Dorsal-fin length | 7.4–12.2 | 9.4 ± 2.5 | 9.1–16.8 | 12.9 ± 2.9 | 12.6–16.5 | 14.5 ± 1.4 | 7.4–14.1 | 10.0 ± 2.6 | 7.7–14.7 | 12.2 ± 2.6 | 14.2–23.8 | 18.6 ± 3.8 | 0.279 |
| Pectoral-fin length | 6.7–11.3 | 8.7 ± 2.3 | 7.7–17.2 | 12.1 ± 3.5 | 11.6–15.1 | 12.9 ± 1.4 | 7.0–14.8 | 9.6 ± 3.0 | 7.1–15.4 | 12.7 ± 3.2 | 13.2–20.4 | 16.6 ± 3.1 | 0.497 |
| Pectoral-fin base length | 1.0–2.7 | 1.7 ± 0.9 | 1.9–5.4 | 3.1 ± 1.2 | 2.3–4.5 | 3.4 ± 0.8 | 1.4–4.4 | 2.7 ± 1.1 | 1.3–2.5 | 2.0 ± 0.5 | 1.8–5.6 | 3.7 ± 1.6 | 0.194 |
| Pre-pectoral length | 7.1–13.4 | 10.2 ± 3.2 | 10.1–21.4 | 15.8 ± 4.3 | 15.6–20.7 | 17.6 ± 1.9 | 8.5–17.0 | 12.7 ± 3.6 | 9.5–20.1 | 15.4 ± 4.1 | 14.6–26.0 | 19.3 ± 4.7 | 0.194 |
| Pelvic-fin length | 4.9–8.7 | 6.9 ± 1.9 | 4.5–13.7 | 8.9 ± 3.4 | 9.4–13.2 | 11.2 ± 1.4 | 5.7–10.7 | 7.6 ± 2.1 | 5.1–11.9 | 8.1 ± 2.5 | 11.1–15.6 | 13.4 ± 2.1 | 0.776 |
| Pelvic-fin base length | 0.9–2.0 | 1.4 ± 0.6 | 1.2–3.3 | 2.1 ± 0.8 | 1.7–3.3 | 2.3 ± 0.5 | 1.2–2.2 | 1.8 ± 0.5 | 1.0–2.1 | 1.7 ± 0.5 | 1.4–3.5 | 2.3 ± 0.8 | 0.497 |
| Pre-pelvic length | 15.0–26.7 | 21.0 ± 5.9 | 21.4–45.9 | 32.7 ± 9.2 | 31.6–44.2 | 37.9 ± 4.9 | 16.9–35.8 | 25.9 ± 7.4 | 17.7–42.7 | 33.2 ± 9.9 | 32.4–52.5 | 40.9 ± 8.3 | 0.279 |
| Anal-fin length | 5.8–9.8 | 7.9 ± 2.0 | 7.1–14.7 | 10.5 ± 2.8 | 10.9–13.2 | 11.8 ± 0.9 | 5.4–11.2 | 7.7 ± 2.2 | 5.4–12.1 | 9.2 ± 2.5 | 11.3–16.2 | 13.6 ± 2.1 | 0.497 |
| Anal-fin base length | 2.9–4.3 | 3.5 ± 0.7 | 3.3–6.9 | 5.2 ± 1.4 | 5.2–8.3 | 6.6 ± 1.2 | 2.2–5.4 | 3.7 ± 1.2 | 2.9–6.5 | 5.2 ± 1.4 | 4.7–7.8 | 6.4 ± 1.5 | 0.194 |
| Pre-anal length | 20.2–37.0 | 28.7 ± 8.4 | 32.2–65.8 | 47.3 ± 12.5 | 48.5–65.7 | 56.0 ± 6.5 | 26.6–51.0 | 38.1 ± 10.2 | 27.3–65.8 | 49.9 ± 14.8 | 48.0–75.7 | 59.5 ± 11.1 | 0.133 |
| Distance between base of pectoral fin and pelvic fin origin | 7.1–11.7 | 9.4 ± 2.3 | 9.7–19.9 | 14.4 ± 4.1 | 13.1–20.6 | 17.4 ± 2.8 | 7.7–15.4 | 11.3 ± 3.0 | 8.1–22.7 | 17.7 ± 5.9 | 14.8–21.8 | 17.6 ± 3.1 | 0.279 |
| Distance between base of pelvic fin and anal fin origin | 4.8–8.4 | 6.6 ± 1.8 | 8.5–17.0 | 12.4 ± 3.0 | 13.4–19.0 | 15.8 ± 1.7 | 8.0–13.8 | 10.5 ± 2.7 | 9.7–23.1 | 16.6 ± 4.9 | 11.3–18.9 | 14.9 ± 2.9 | 0.048 |
| Distance between anal fin and posterior margin of anus | 0.4–1.6 | 1.2 ± 0.7 | 1.2–2.7 | 2.1 ± 0.6 | 0.8–3.8 | 2.2 ± 1.0 | 0.6–2.1 | 1.3 ± 0.5 | 0.5–1.1 | 0.9 ± 0.3 | 0.2–2.6 | 0.9 ± 1.0 | 0.194 |
| Caudal peduncle length | 5.1–9.4 | 6.9 ± 2.3 | 6.7–12.3 | 9.7 ± 2.2 | 9.0–14.4 | 12.0 ± 2.1 | 5.5–10.4 | 7.7 ± 1.9 | 6.1–14.5 | 11.4 ± 3.3 | 10.9–16.6 | 14.0 ± 2.6 | 0.279 |
| Caudal peduncle depth | 2.6–4.6 | 3.5 ± 1.0 | 4.1–8.3 | 6.0 ± 1.6 | 4.0–6.5 | 5.4 ± 0.9 | 3.3–6.0 | 4.5 ± 1.1 | 2.9–6.5 | 5.3 ± 1.5 | 4.3–8.4 | 5.8 ± 1.6 | 0.194 |
| Maxillary barbel length | 1.6–4.7 | 2.7 ± 1.7 | 3.7–9.2 | 6.3 ± 2.0 | 4.5–7.9 | 6.1 ± 1.4 | 2.8–8.3 | 5.0 ± 2.2 | 0.9–1.8 | 1.4 ± 0.4 | 7.6–12.0 | 9.9 ± 1.9 | 0.085 |
| Inrostral barbel length | 1.2–3.3 | 2.0 ± 1.2 | 1.7–5.7 | 3.8 ± 1.5 | 3.3–5.0 | 4.0 ± 0.5 | 2.2–4.5 | 3.4 ± 1.1 | 1.3–2.8 | 2.2 ± 0.6 | 4.1–8.6 | 6.5 ± 2.0 | 0.133 |
| Outrostral barbel length | 2.1–4.9 | 3.4 ± 1.4 | 4.1–10.8 | 7.5 ± 2.5 | 6.1–10.2 | 7.8 ± 1.4 | 3.4–8.3 | 5.5 ± 1.9 | 3.0–6.7 | 5.1 ± 1.4 | 10.2–15.6 | 12.6 ± 2.3 | 0.085 |

Next page

Continued

|  | *TC* vs. *TP* | *TC* vs. *TA* | *TC* vs. T*B* | *TC* vs. *TZ* | *TR* vs. *TP* | *TR* vs. *TA* | *TR* vs. *TB* | *TR* vs. *TZ* | *TP* vs. *TA* | *TP* vs. *TB* | *TP* vs. *TZ* | *TA* vs. *TB* | *TA* vs. *TZ* |
| --- | --- | --- | --- | --- | --- | --- | --- | --- | --- | --- | --- | --- | --- |
| Total length | 0.018 | 0.548 | 0.143 | 0.057 | 0.321 | 0.282 | 0.724 | 0.171 | 0.012 | 0.518 | 0.797 | 0.177 | 0.030 |
| Standard length | 0.145 | 0.381 | 0.143 | 0.057 | 0.167 | 0.414 | 0.622 | 0.171 | 0.008 | 0.699 | 1.000 | 0.177 | 0.052 |
| Body depth | 0.209 | 0.262 | 0.250 | 0.057 | 0.743 | 0.282 | 0.943 | 0.222 | 0.181 | 0.898 | 0.298 | 0.329 | 0.017 |
| Body width | 0.036 | 0.381 | 0.250 | 0.057 | 0.963 | 0.108 | 0.622 | 0.833 | 0.456 | 1.000 | 0.364 | 0.247 | 0.126 |
| Head length | 0.100 | 0.381 | 0.250 | 0.057 | 0.423 | 0.573 | 0.833 | 0.127 | 0.113 | 0.112 | 0.240 | 0.537 | 0.052 |
| Head depth | 0.100 | 0.381 | 0.250 | 0.114 | 1.000 | 0.755 | 0.622 | 0.435 | 0.456 | 0.606 | 0.518 | 0.931 | 0.126 |
| Head width | 0.036 | 0.381 | 0.393 | 0.114 | 0.370 | 0.491 | 0.284 | 0.435 | 0.224 | 0.042 | 1.000 | 0.931 | 0.126 |
| Pre-anterior nostril length | 0.036 | 0.714 | 0.786 | 0.057 | 0.888 | 0.345 | 0.093 | 0.622 | 0.145 | 0.007 | 0.898 | 0.429 | 0.177 |
| Distance between anterior nostrils | 0.209 | 0.548 | 0.571 | 0.114 | 0.815 | 0.662 | 0.093 | 0.833 | 0.224 | 0.004 | 0.898 | 0.126 | 0.247 |
| Distance between posterior nostrils | 0.018 | 0.167 | 0.071 | 0.036 | 0.963 | 1.000 | 0.093 | 0.171 | 0.776 | 0.083 | 0.060 | 0.052 | 0.126 |
| Distance between anterior and posterior nostrils | 0.209 | 0.381 | 0.036 | 0.057 | 0.059 | 0.282 | 0.002 | 0.002 | 0.026 | 0.001 | 0.001 | 0.004 | 0.030 |
| Snout length | 0.036 | 0.714 | 0.786 | 0.114 | 0.277 | 0.755 | 0.724 | 0.222 | 0.113 | 0.060 | 0.699 | 0.931 | 0.052 |
| Upper jaw length | 0.018 | 0.381 | 0.393 | 0.114 | 0.888 | 0.345 | 0.284 | 0.833 | 0.113 | 0.112 | 0.699 | 1.000 | 0.126 |
| Lower jaw length | 0.018 | 0.095 | 0.250 | 0.036 | 0.815 | 0.573 | 0.833 | 0.724 | 0.272 | 0.518 | 1.000 | 0.792 | 0.329 |
| Mouth width | 0.064 | 0.381 | 0.071 | 0.036 | 0.888 | 0.228 | 0.222 | 0.622 | 0.113 | 0.240 | 0.298 | 0.052 | 0.052 |
| Eye diameter | 0.009 | 0.024 | 0.036 | 0.036 | 0.888 | 0.003 | 0.171 | 0.006 | 0.001 | 0.364 | 0.004 | 0.030 | 0.662 |
| Interorbital distance | 0.145 | 0.548 | 0.393 | 0.400 | 0.606 | 0.852 | 1.000 | 1.000 | 0.776 | 0.606 | 0.364 | 0.931 | 0.792 |
| Predorsal length | 0.036 | 0.548 | 0.143 | 0.036 | 0.370 | 0.414 | 0.833 | 0.222 | 0.066 | 0.606 | 0.699 | 0.247 | 0.052 |
| Dorsal-fin base length | 0.036 | 0.714 | 0.143 | 0.036 | 0.114 | 0.491 | 0.622 | 0.030 | 0.008 | 0.240 | 0.298 | 0.247 | 0.009 |
| Dorsal-fin length | 0.036 | 1.000 | 0.143 | 0.036 | 0.321 | 0.345 | 1.000 | 0.019 | 0.026 | 0.147 | 0.029 | 0.177 | 0.004 |
| Pectoral-fin length | 0.018 | 0.905 | 0.143 | 0.036 | 0.673 | 0.414 | 0.435 | 0.030 | 0.036 | 0.364 | 0.012 | 0.247 | 0.017 |
| Pectoral-fin base length | 0.100 | 0.381 | 0.786 | 0.140 | 0.370 | 1.000 | 0.284 | 0.354 | 0.328 | 0.029 | 0.518 | 0.247 | 0.329 |
| Pre-pectoral length | 0.018 | 0.381 | 0.250 | 0.036 | 0.606 | 0.414 | 0.943 | 0.222 | 0.113 | 0.797 | 0.606 | 0.247 | 0.052 |
| Pelvic-fin length | 0.036 | 0.714 | 0.786 | 0.036 | 0.139 | 0.852 | 1.000 | 0.019 | 0.026 | 0.083 | 0.029 | 0.792 | 0.004 |
| Pelvic-fin base length | 0.145 | 0.262 | 0.393 | 0.140 | 0.541 | 0.755 | 0.833 | 0.524 | 0.181 | 0.112 | 0.898 | 0.537 | 0.247 |
| Pre-pelvic length | 0.036 | 0.548 | 0.143 | 0.036 | 0.481 | 0.414 | 0.622 | 0.222 | 0.050 | 0.606 | 0.438 | 0.177 | 0.030 |
| Anal-fin length | 0.036 | 0.714 | 0.571 | 0.036 | 0.236 | 0.282 | 0.833 | 0.045 | 0.012 | 0.083 | 0.112 | 0.429 | 0.004 |
| Anal-fin base length | 0.018 | 0.905 | 0.143 | 0.036 | 0.093 | 0.228 | 0.724 | 0.127 | 0.008 | 0.298 | 1.000 | 0.126 | 0.017 |
| Pre-anal length | 0.018 | 0.262 | 0.143 | 0.036 | 0.236 | 0.491 | 0.524 | 0.127 | 0.026 | 0.797 | 0.606 | 0.177 | 0.030 |
| Distance between base of pectoral fin and pelvic fin origin | 0.018 | 0.548 | 0.143 | 0.036 | 0.139 | 0.282 | 0.127 | 0.171 | 0.026 | 0.518 | 0.699 | 0.082 | 0.017 |
| Distance between base of pelvic fin and anal fin origin | 0.009 | 0.095 | 0.036 | 0.036 | 0.046 | 0.414 | 0.093 | 0.127 | 0.008 | 0.518 | 0.898 | 0.030 | 0.052 |
| Distance between anal fin and posterior margin of anus | 0.373 | 0.905 | 0.571 | 0.790 | 0.888 | 0.142 | 0.011 | 0.065 | 0.224 | 0.029 | 0.042 | 0.177 | 0.247 |
| Caudal peduncle length | 0.036 | 0.548 | 0.143 | 0.036 | 0.093 | 0.228 | 0.222 | 0.019 | 0.018 | 0.898 | 0.112 | 0.082 | 0.004 |
| Caudal peduncle depth | 0.145 | 0.262 | 0.143 | 0.071 | 0.606 | 0.282 | 1.000 | 0.724 | 0.272 | 0.797 | 0.797 | 0.429 | 0.247 |
| Maxillary barbel length | 0.064 | 0.167 | 0.071 | 0.036 | 0.963 | 0.491 | 0.002 | 0.011 | 0.456 | 0.001 | 0.002 | 0.004 | 0.009 |
| Inrostral barbel length | 0.018 | 0.167 | 0.786 | 0.036 | 0.481 | 0.950 | 0.171 | 0.030 | 0.607 | 0.004 | 0.012 | 0.082 | 0.017 |
| Outrostral barbel length | 0.018 | 0.167 | 0.250 | 0.036 | 0.888 | 0.282 | 0.284 | 0.006 | 0.113 | 0.029 | 0.001 | 0.931 | 0.004 |
